# Norgal: extraction and de novo assembly of mitochondrial DNA from whole-genome sequencing data

**DOI:** 10.1186/s12859-017-1927-y

**Published:** 2017-11-21

**Authors:** Kosai Al-Nakeeb, Thomas Nordahl Petersen, Thomas Sicheritz-Pontén

**Affiliations:** Department of Bio and Health Informatics, Technical University of Denmark, Kemitorvet, Building 208, Kgs Lyngby, 2800 Denmark

**Keywords:** Mitochondrial dna, K-mer, Next-generation sequencing, De novo assembly

## Abstract

**Background:**

Whole-genome sequencing (WGS) projects provide short read nucleotide sequences from nuclear and possibly organelle DNA depending on the source of origin. Mitochondrial DNA is present in animals and fungi, while plants contain DNA from both mitochondria and chloroplasts. Current techniques for separating organelle reads from nuclear reads in WGS data require full reference or partial seed sequences for assembling.

**Results:**

Norgal (de Novo ORGAneLle extractor) avoids this requirement by identifying a high frequency subset of k-mers that are predominantly of mitochondrial origin and performing a de novo assembly on a subset of reads that contains these k-mers. The method was applied to WGS data from a panda, brown algae seaweed, butterfly and filamentous fungus. We were able to extract full circular mitochondrial genomes and obtained sequence identities to the reference sequences in the range from 98.5 to 99.5%. We also assembled the chloroplasts of grape vines and cucumbers using Norgal together with seed-based de novo assemblers.

**Conclusion:**

Norgal is a pipeline that can extract and assemble full or partial mitochondrial and chloroplast genomes from WGS short reads without prior knowledge. The program is available at: https://bitbucket.org/kosaidtu/norgal.

**Electronic supplementary material:**

The online version of this article (doi:10.1186/s12859-017-1927-y) contains supplementary material, which is available to authorized users.

## Background

Certain organelles such as mitochondria have their own distinct genomes. The mitochondrial genome - the mitogenome - differs significantly from eukaryotic nuclear genomes e.g. by typically being circular and smaller in size [1]. The mitogenome can be sequenced experimentally by isolating the mitochondria, amplifying the mitochondrial DNA (mtDNA) with PCR using primers from mtDNA of closely related organisms and sequencing the PCR products. With high-throughput whole-genome sequencing (WGS), the data typically contains mitochondrial DNA in addition to nuclear DNA and does not require the isolation of mitochondria before-hand. This makes WGS data a valuable resource for extracting and assembling mitogenomes, and can potentially replace targeted sequencing.

Current methods to extract mtDNA from WGS data require a short seed sequence to initiate assembly [2, 3]. However, for unknown organisms whose mitogenomes differ significantly from the currently known mitogenomes, this can be inconvenient and challenging. To avoid this problem, we developed a reference-independent method based on k-mer frequencies that takes advantage of mitochondria being present 10-100 times more in a cell than the nucleus [4].

This means that in sequencing experiments the mitogenome will have a higher read depth compared to the nuclear genome and this difference in the read depth levels can be used to separate the reads into two groups; those of nuclear and those of mitochondrial origin.

The separation of the two types of reads is done by counting occurrences of subsequences of length k in the reads - k-mers - and classifying reads that have k-mers that are found more times than the nuclear read depth as being of non-nuclear origin. These non-nuclear reads with k-mers above the nuclear read depth threshold may come from the mitochondria and plastids or from certain regions in the nuclear genome such as repeats, NUMT’s etc. The predominantly mitochondrial reads can then be de novo assembled into non-nuclear sequences where it is reasonable to assume that the longest contig in this assembly would be from mitochondria or plastids as the longer nuclear genome would not be assembled. Norgal is our implementation of this assembly method and provides annotation and evaluation of the final sequence. In the case where an assembly is partial or fragmented, the user can use this sequence as a reference for one of the current reference-based extraction tools. Recently, the mitochondrial genome of the Oriental hornet (Vespa orientalis) was published using a Norgal assembly [5].

## Implementation

**Fig. 1:**
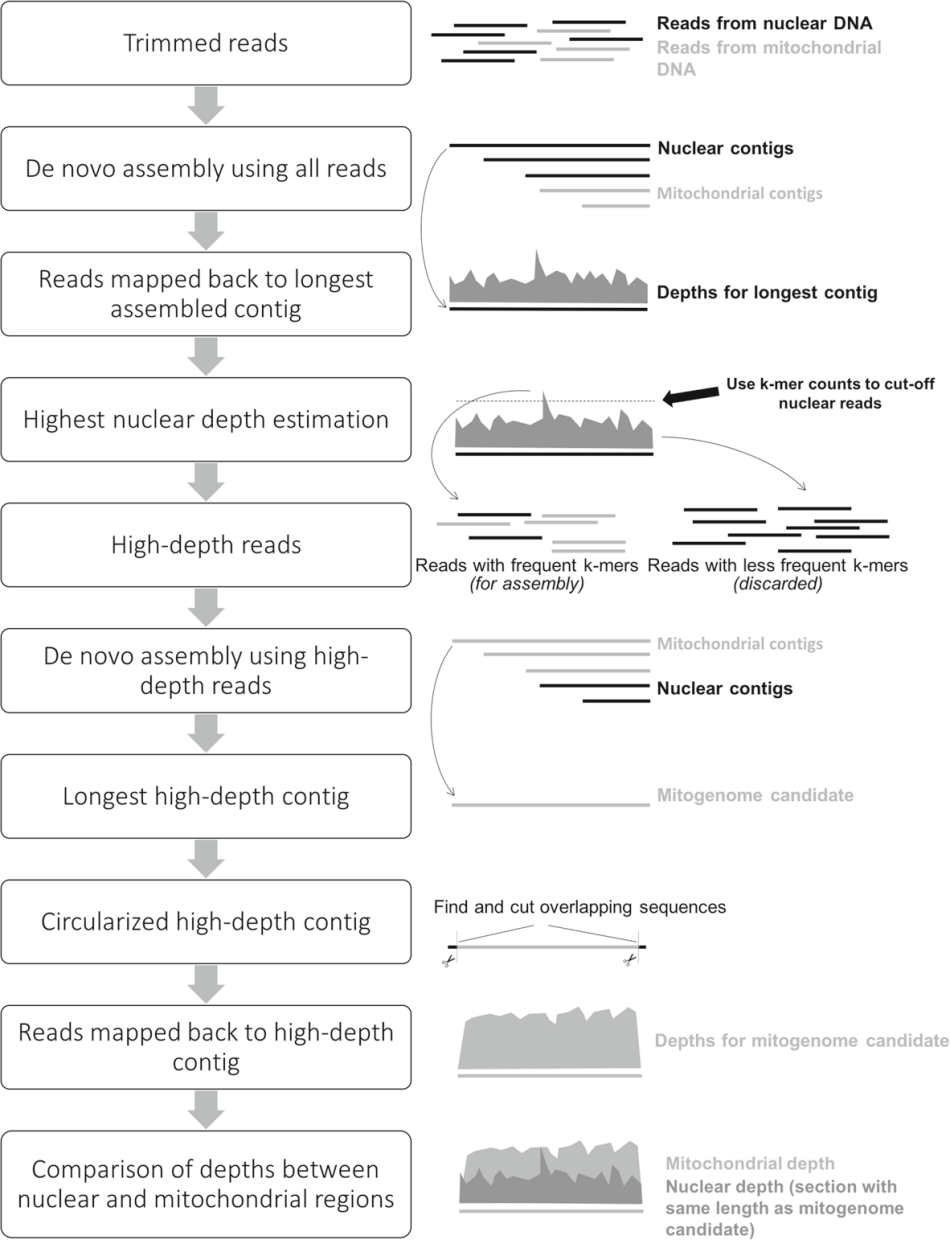
Workflow of Norgal. This diagram shows how Norgal seperates mitochondrial reads from nuclear reads and assembles the mitochondrial reads into a partial or complete mitogenome

Norgal uses raw short NGS reads from WGS data as input and outputs either a full or partial mitogenome. Norgal is written in python3 but is backwards compatible with python2.7 and requires java and the python library matplotlib for plotting. It relies on a range of bundled software for the different steps in the pipeline. Figure 1 shows the workflow of Norgal which has the following steps:

1. Trim and remove adapters from NGS reads using *AdapterRemoval* [6] and perform a de novo assembly using *MEGAHIT* [7].
2. Map the reads back to the longest assembled sequence using *bwa mem* [8] and calculate the read depths for each position in order to determine the nuclear depth threshold (ND threshold).
3. Count kmers of size 31 in all reads and only keep a subset of reads that contains at least one 31-kmer with a frequency that is greater than the ND threshold. This is done using the program *BBTools* [9].
4. Perform a de novo assembly using *idba_ud* [10] with the reads containing the frequent kmers and extract either the longest contig or optionally the longest contig with a predicted cytochrome c oxidase subunit 1 (COI) gene.
5. Examine circularity of the longest contig, determine read depth, identify potential mitochondrial and chloroplast contigs, and output plots comparing depths between this contig and the longest contig from the assembly in step (1).

These steps are explained in more details in the following sections.

### Pre-processing reads

Raw reads may contain non-biological DNA sequences from the sequencing process, such as adapter and primer sequences. If these are not removed before-hand, Norgal removes adapters and trims NGS reads using *AdapterRemoval* with --minlength 30 and default settings.

### Estimating nuclear read depth threshold

If no reference sequence from the nuclear genome is provided, an initial de novo assembly is performed using the program *MEGAHIT* with default settings and the k-mer range: 21, 49, 77 and 105. Norgal assumes that the longest assembled sequence (contig) is nuclear. The reads are then mapped back to the longest assembled contig using *bwa mem* with default settings. If the longest assembled contig is longer than 100,000 base pairs, only the first 100,000 base pairs are used as it should be enough to determine the depth. The read depths of the mapped reads to this contig are used to determine the nuclear depth threshold (ND threshold) which is defined as the mean of all non-zero read depths from the 25^*th*^ to the 75^*th*^ percentile range multiplied with five:

1 $$  \text{ND threshold} = 5\cdot \frac{\sum_{i=25^{th} percentile}^{75^{th} percentile}d_{i}}{n}  $$

Here, *d*_*i*_ is the read depth at index *i* in a sorted array of non-zero read depths from the the longest assembled contig and *n* is the number of non-zero read depths in the percentile range. If all read depths are non-zero, *n* is half of the length of the contig.

The mitochondrial copy numbers have previously been determined to be in the range of 10 to 100 times higher than the nuclear read depth [4]. Norgal uses the multiplication factor 5 in Eq. (1) as it lies between the lowest reported number of mitochondria in the literature and the nuclear depth. This threshold can be set manually by the user and should be slightly higher than the depth.

### Binning reads based on k-mer occurrences

There is a direct correlation between genome depth and k-mer counts (also called k-mer depths) [11]:

2 $$  N = M\cdot \frac{L}{L-k+1},\text{where k \(<\) L+1}  $$

where *N* is the genome depth, *M* is the k-mer depth, *L* is the read length and *k* is the k-mer size.

While it may not be feasible to determine the depth over each read, it is much less computationally intensive to determine which k-mers are present in each read and how often these k-mers are found in the total read pool and then translating this to read depth. This can be done because the number of times a k-mer is found in the total read pool corresponds to the k-mer depth, *M*, in the above Eq. (2). Since the kmer size, *k*, is known before-hand and the read length, *L*, can be determined effortlessly, it is straight-forward to calculate the genomic depth, *N*, of the region from which the read originated if *M* is known. However, depending on the k-mer size, it is reasonable to assume that k-mers are not unique to the genomic region they are found in, and thus the calculated genomic depth may be overestimated. Binning reads based on the estimated read depths using this equation may therefore result in *false positive* mitochondrial reads, i.e. reads from the nuclear genome binned as mitochondrial reads. This may lead to a number of small nuclear contigs in the mitochondrial assembly.

When the k-mer counts in the read pool have been calculated, the reads that come from genomic regions with depths above the ND threshold can be identified and extracted using the above Eq. (2). The counting and binning can be done by the program *BBTools*. As the number of k-mers in a read pool can be very large and may not fit into computer memory, *BBTools* instead stores the k-mers in a probabilistic data structure called a Count-Min Sketch (CMS) invented in 2004 [12] which is based on a set of bit-arrays and hash-functions. *BBTools*’s implementation of CMS can keep track of k-mers and their counts, but may overestimate some k-mer depths because of possible hash collisions, which as mentioned before may lead to small nuclear contigs in the assembly.

In Norgal’s usage scenario it is acceptable not to discard reads with non-frequent k-mers (nuclear reads - false positives) as these will only result in small contigs. On the other hand, it is not acceptable to discard reads with frequent k-mers (mitochondrial reads - false negatives) as this may lead to a partial mitochondrial assembly. This makes a CMS optimal for this problem as it can only be inaccurate when overestimating k-mer counts. This means that no reads with a higher read depth than the threshold can be discarded.

### Assembly with high-frequency k-mers

The binned reads with high-frequency k-mers are used for an assembly with *idba_ud* with default settings which does multiple assemblies with different k-mer sizes in the range: 20, 40, 60, 80 and 100. This second assembly only contains contigs that have a high read-depth of at least the ND threshold.

### Annotation and validation

The contigs are sorted after length and per default the longest contig is extracted. Another option is to select the longest contig that has the best hits to full RefSeq mitochondrial or pastid genomes. The extracted contig is tested for circularity by comparing the ends of the contig and finding overlaps. Any overlapping base pairs are cut and the final sequence is reported as a potential mtDNA candidate. The reads are mapped back to this potential mtDNA sequence and Norgal outputs a graph with the read depths as well as the read depths of a section of the nuclear DNA (the assembled longest contig from the first assembly) spanning the same length as the mtDNA candidate. This graph with the two sets of read depths may be used for validation of the mtDNA candidate, so if the depths over the mtDNA candidate is around 10-100 higher than the depths over the nuclear region, it increases the evidence that the candidate is from the mitogenome.

Norgal searches the full assembly for both complete mitochondrial and plastid genomes using BLAST [13, 14] with default values and reports the best 10 hits sorted by bit-score.

## Results and discussion

Twenty WGS datasets were downloaded from the Short Read Archive (SRA) (ncbi.nlm.nih.gov/sra). The results of Norgal on these datasets can be seen in the Additional file 1: Section S4. Norgal extracted and assembled the full circular mitogenomes in 10 of the 20 cases, while only partially assembling the mitogenomes (and chloroplasts) for the rest, ranging from 1–49% coverage.

Table 1 shows the reports that Norgal outputs for a subset of the datasets. It shows that the longest contig is usually the mitochondrial or plastid genome.

**Table 1:** Norgal BLAST output for a subset of the datasets

Organism	Type	Scaffold:Scaffold-length	Identity	Align. length	Ref. length	E-value	Bit-score	Best-hit reference
A. melanoleuca	m	scaffold_0:16876	99.54	16181	16805	0	29438	Ailuropoda melanoleuca mitochondrion
S. japonica	m	scaffold_0:37756	100	35932	37654	0	66354	Saccharina sp. ye-C12 mitochondrion
P. glaucus	m	scaffold_0:15378	100	7814	15306	0	14430	Papilio glaucus mitochondrion
A. niger	m	scaffold_0:31289	99.12	9284	31103	0	16661	Aspergillus niger mitochondrion
P. papatasi	m	scaffold_0:15338	99.54	14927	15557	0	27180	Phlebotomus papatasi mitochondrion

Note how the best hit for each organisms is always scaffold_0 which is also the longest scaffold in the assembly. A full table of the 10 best hits for each organisms can be found in the Additional file 1: Section S1

The assembled mitogenomes were generally highly similar to the reference sequences, though rearrangements of shorter sequences, especially in the hypervariable regions of the control regions [15], were occasionally observed.

### Comparison with current methods

Norgal was benchmarked against two other tools, MITOBim and NOVOPlasty, which both require at least a seed sequence to initiate an assembly. To our knowledge, there is no current tool that can assemble mitogenomes completely independently of reference or seed sequences. Both MITObim and NOVOPlasty can use relatively small sequences as a seed, such as a single gene sequence from the target mitogenome or from a more distantly related organism. In comparison, Norgal requires no seed or reference sequence and relies solely on differential k-mer frequencies in the reads which it automatically detects to de novo assemble the mitogenome. Table 2 shows the performance of the three tools on a subset of the tested datasets spanning different eukaryote organism groups. The benchmark was run on a computer cluster node with 4 CPU’s and 120 GB of memory. The accuracy was comparable among all three methods and they all produced full circular mitochondrial genomes that covered the reference sequence entirely.

**Table 2:** Benchmarking of Norgal and comparison with MITOBim and NOVOPlasty

	Norgal		MITOBim v1.9		NOVOPlasty v2.6.2	
Organism	Identity to reference sequence	Input	Identity to reference sequence	Input	Identity to reference sequence	Input
*A. melanoleuca* (Giant Panda)	*99.5*%	Raw reads	98.8%	Trimmed and interleaved reads, reference mitogenome (NC_009492.1)	99.1%	Raw reads, insert size, read length, reference COI sequence (DQ093081.1)
*S. japonica* (Japanese Seaweed)	*99.8*%	Raw reads	99.0%	Trimmed and interleaved reads, reference mitogenome (NC_013476.1)	*99.8*%	Raw reads, mitogenome size range, insert size, read length, reference COI sequence (JN873222.1)
*P. glaucus* (Swallowtail butterfly)	99.8%	Raw reads	98.5%	Trimmed and interleaved reads, reference mitogenome (KR822739.1)	*100.0*%	Raw reads, insert size, read length, reference COI sequence (KT286455.1)
*A. niger*	98.7%	Raw reads	97.8%	Trimmed and interleaved reads, reference mitogenome (NC_007445.1)	*98.9*%	Raw reads, mitogenome size range, insert size, read length, reference COI sequence (EF180096.1)
*P. papatasi* (Sand fly)	98.5%	Raw reads	99.0%	Trimmed and interleaved reads, reference mitogenome (NC_028042.1)	*99.9*%	Raw reads, insert size, read length, reference COI sequence (KU659597.1)

The full results of the benchmark can be seen in the Additional file 1: Section S3

The reference sequences were determined by mapping the reads to the NCBI references and correcting the nucleotide differences

The highest identity scores are italicized

The peak memory usage was 38-48 GB for Norgal, 1-13 GB for MITOBim and 33-53 GB for NOVOPlasty.

In terms of runtime Norgal is the slowest by using nine hours on average to assemble the mitogenome. MITOBim used three hours on average while NOVOPlasty only used half an hour. These runtimes exclude the time for preparing the input data for the programs. The reason Norgal is slower is because of the initial full assembly and mapping that determines the nuclear depth. This part consists of multiple assemblies of the whole read pool with a range of different k-mers. If a subsequence of the nuclear genome or the depth of coverage is given to Norgal, the runtime decreases significantly.

Regarding ease of use, all programs run on the command line. Norgal requires the path to the raw reads and a name for the output folder. MITOBim can run in several modes including a 2-step mode where an initial assembly with the program MIRA is used as input. The mode used in this comparison requires only trimmed and interleaved reads as input as well as the seed sequence. NOVOPlasty uses a single configuration file as input which can be modified with the different input parameters such as the path to a reference or seed sequence.

In short, Norgal does not require a reference or short seed sequences compared to MITOBim and NOVOPlasty while still achieving similar accuracy. However, both MITOBim and NOVOPlasty are significantly faster and use less resources.

### Extraction of plastid DNA using a 2-step procedure

Plants have long mitogenomes compared to e.g. vertebrates [16] and additionally have chloroplasts genomes which are present in high copy numbers [17]. An assembly of reads with highly frequent k-mers would most likely contain fragmented chloroplast and mitochondrial contigs. Norgal saves the assembly made from the reads with highly frequent k-mers in addition to the extracted mitogenome candidate and a report with best BLAST-hits. Contigs from this assembly can be used as the input seed sequence for current plastid assembly programs such as MITOBim and NOVOPlasty. This can be relevant in projects involving a large number of diverse and unknown organisms. Norgal’s output can in this scenario be used to automatically select relevant seeds for a further assembly.

This approach was tried with a fragmented assembly of the grape plant from Norgal and then using NOVOPlasty v1.1 on the longest contigs. The second-longest contig resulted in the full chloroplast genome with an identity of 98% to the reference sequence and a combined runtime of 12 h (see Additional file 1: Section S2).

The approach was also tested on a cucumber sample. Cucumbers have large mitogenomes that are split into three separate chromosomes. Norgal outputted a series of contigs from the chloroplasts and mitochondria. The chloroplast contig was used as a seed sequence for NOVOPlasty and resulted in the full cucumber chloroplast genome with 100% identity to the reference chloroplast.

For users interested in completely unknown chloroplast or other organelle genomes for which there are no known sequences, the following approach is suggested:

1. Extract contigs of interests from the Norgal assembly, such as the ten longest contigs or the contigs with hits from the BLAST-search
2. Run MITOBim or NOVOPlasty or another assembler that can extend seed sequences on each of the ten contigs
3. Validate the output by:
  - mapping reads back to the contigs and compare depths to the nuclear depth
  - checking for circularity in the contigs
  - annotating the contigs with relevant features e.g. mitochondrial genes etc.

### Assembly complications

As Norgal is based on differences in k-mer frequencies it is not suited for metagenomics datasets or datasets where the reads are evenly distributed across the mitogenome and nuclear genome (for example organisms with low copy numbers of mitochondria or samples with many PCR duplicates). This might result in fragmented assemblies as seen in the grape and cucumber case, where the longest assembled scaffolds were partial sequences of the mitochondria or chloroplast. This also means that Norgal in general requires a high depth of coverage in order to accurately separate the reads.

The nuclear genome can have sequences of mitochondrial origin (NUMTs) which are not part of the mitogenome [18]. As Norgal counts k-mers in reads it may include reads from those NUMT regions, as reads that come from these regions may share k-mers with reads from similar regions in the mitogenome. They will consequently not be discarded before assembly and may be incorporated in the final assembled mitogenome sequence. This is undesirable and a BLAST search with some of the assembled mitogenomes against the nuclear genomes did suggest that they had incorporated some NUMT sequences.

As de novo assemblers based on De Bruijn graphs can theoretically struggle with repeat regions that span the insert size of read libraries [10], such a case may lead to fragmented assemblies when using paired end reads with short insert sizes.

Irregular and complex mitochondria (e.g. cucumber mitochondrial genomes that are split into multiple chromosomes, one of which is very long) may further complicate assembly. Some organisms have fewer mitochondria in their cells compared to what is expected from the litterature. This would require setting the depth cut-off manually instead of using the ND threshold.

## Conclusion

Norgal is a tool for extracting mitochondrial DNA from WGS data, especially in situations where reference sequences are unavailable. Plastid genomes were assembled using a proposed 2-step procedure that uses Norgal output as a seed to existing plastid assemblers. Nogal’s success with the 2-step procedure shows that Norgal is optimal in scenarios where the mitochondrial genome is completely unknown and cannot be assembled from any known reference or seed sequences. This tool contributes to the field of discovering and assembling novel mitochondrial sequences from WGS data.

## Availability and requirements

The datasets analysed during the current study are available in the NCBI SRA repository, https://www.ncbi.nlm.nih.gov/sraunder the following accession numbers: SRR1801279, SRR2089773, SRR2089774, SRR2089775, SRR1707287, SRR543219, SRR1997462, SRR2015301, SRR899957, SRR1291041, SRR958464, SRR504904, SRR942310, SRR1993099, ERR1437502, ERR771129, SRR2984940, SRR494422, SRR494432, and SRR2043182.

**Project name:** Norgal

**Project home page:** https://bitbucket.org/kosaidtu/norgal

**Archived version:** https://github.com/kosaidtu/norgal/releases/download/v1.0/norgal.tar

**Operating system(s):** Linux

**Programming language:** Python3

**Other requirements:** bash, java, matplotlib (python3 package)

**License:** MIT License (BBTools is copyrighted to The Regents of the University of California, through Lawrence Berkeley National Laboratory.

**Any restrictions to use by non-academics:** MIT License

## Additional file

Additional file 1 A.docx-document with full results and detailed benchmarking between Norgal and MITOBim and NOVOPlasty. Section S1: Full Norgal output of subset of test data. Section S2: Extraction of chloroplast from Vittis vinifera (Grape vine). Section S3: Benchmarking against other methods. Section S4: Mitochondrial test data sets. (DOCX 1485 kb)

## Abbreviations

BLAST: Basic local alignment search tool
bp: Base pairs
DNA: Deoxyribonucleic acid
k-mer: DNA subsequence of length k
mitogenome: Mitochondrial genome
mtDNA: Mitochondrial DNA
ND threshold: Nuclear depth threshold
NGS: Next-generation sequencing
NUMTs: Nuclear mitochondrial DNA segment
PCR: Polymerase chain reaction
WGS: Whole-genome sequencing
