# Supplementary material for: Norgal: extraction and de novo assembly of mitochondrial DNA from whole-genome sequencing data
Source: BMC Bioinformatics. 2017 Nov 21;18:510. doi: 10.1186/s12859-017-1927-y (PMC5699183; doi:10.1186/s12859-017-1927-y)
Supplement: Additional file 1 — A.docx-document with full results and detailed benchmarking between Norgal and MITOBim and NOVOPlasty. Section S1: Full Norgal output of subset of test data. Section S2: Extraction of chloroplast from Vittis vinifera (Grape vine). Section S3: Benchmarking against other methods. Section S4: Mitochondrial test data sets. (DOCX 1485 kb) [file 12859_2017_1927_MOESM1_ESM.docx]

SUPPLEMENTARY MATERIALS

Table of Contents

[1 Full Norgal output of subset of test data 1](#_Toc483238040)

[2 Extraction of chloroplast from Vittis vinifera (Grape vine) 3](#_Toc483238041)

[3 Benchmarking against other methods 4](#_Toc483238042)

[4 Mitochondrial test data sets 6](#_Toc483238043)

# Additional file 1: Section S1. Full Norgal output of subset of test data

Table 1. Norgal output of test data. Note how the best hit is always scaffold_0 which is the longest assembled contig.

| **Organism** | **Type** | **Scaffold:Scaffold-length** | **Identity** | **Align. length** | **Ref. length** | **E-value** | **Bit-score** | **mtRNA-genes** | **Best-hit reference** |
| --- | --- | --- | --- | --- | --- | --- | --- | --- | --- |
| A. melanoleuca | m | scaffold_0:16876 bp | 99.54 | 16181 | 16805 | 0 | 29438 | 26 | Ailuropoda melanoleuca mitochondrion, complete genome |
| A. melanoleuca | p | scaffold_31:1669 bp | 96.34 | 82 | 156073 | 3.00E-29 | 134 | 0 | Nicotiana otophora chloroplast, complete genome |
| A. melanoleuca | m | scaffold_1978:401 bp | 76.76 | 241 | 17056 | 2.00E-26 | 122 | 0 | Dasypus novemcinctus mitochondrion, complete genome |
| A. melanoleuca | p | scaffold_433:693 bp | 96.92 | 65 | 150510 | 2.00E-22 | 110 | 0 | Dracocephalum palmatum chloroplast, complete genome |
| A. melanoleuca | m | scaffold_5460:301 bp | 90.12 | 81 | 16545 | 4.00E-21 | 104 | 0 | Dremomys rufigenis mitochondrion, complete genome |
| S. japonica | m | scaffold_0:37756 bp | 100 | 35932 | 37654 | 0 | 66354 | 31 | Saccharina sp. ye-C12 mitochondrion, complete genome |
| S. japonica | p | scaffold_2:18227 bp | 99.99 | 18227 | 130584 | 0 | 33654 | 8 | Saccharina japonica chloroplast, complete genome |
| S. japonica | p | scaffold_16:10403 bp | 93.77 | 9926 | 130383 | 0 | 14813 | 5 | Undaria pinnatifida chloroplast, complete genome |
| S. japonica | p | scaffold_17:10365 bp | 94.14 | 5254 | 129947 | 0 | 7974 | 3 | Costaria costata chloroplast, complete genome |
| S. japonica | p | scaffold_94:6199 bp | 94.19 | 5111 | 124592 | 0 | 7716 | 5 | Sargassum thunbergii plastid, complete genome |
| S. japonica | p | scaffold_23:9192 bp | 79.68 | 9272 | 138815 | 0 | 6525 | 1 | Pleurocladia lacustris strain Sa2 chloroplast, complete genome |
| S. japonica | p | scaffold_75:6729 bp | 89.32 | 2491 | 124986 | 0 | 3123 | 2 | Fucus vesiculosus chloroplast, complete genome |
| S. japonica | p | scaffold_68:7057 bp | 84.4 | 2590 | 139954 | 0 | 2488 | 5 | Ectocarpus siliculosus chloroplast, complete genome |
| S. japonica | p | scaffold_81:6616 bp | 85.99 | 2191 | 124450 | 0 | 2309 | 2 | Coccophora langsdorfii plastid, complete genome |
| S. japonica | p | scaffold_103:10538 bp | 80.45 | 2655 | 124068 | 0 | 1999 | 5 | Sargassum horneri chloroplast, complete genome |
| P. glaucus | m | scaffold_0:15378 bp | 100 | 7814 | 15306 | 0 | 14430 | 26 | Papilio glaucus mitochondrion, complete genome |
| P. glaucus | m | scaffold_87:1518 bp | 90.18 | 1518 | 15622 | 0 | 1978 | 1 | Ochlodes venata mitochondrion, complete genome |
| P. glaucus | m | scaffold_607:1057 bp | 95.08 | 1056 | 15338 | 0 | 1663 | 1 | Lerema accius mitochondrion, complete genome |
| P. glaucus | m | scaffold_3960:711 bp | 90.14 | 700 | 15530 | 0 | 909 | 0 | Erynnis montanus mitochondrion, complete genome |
| P. glaucus | m | scaffold_2754:773 bp | 87.76 | 768 | 15267 | 0 | 896 | 0 | Potanthus flavus voucher PF13113 mitochondrion, complete genome |
| P. glaucus | m | scaffold_4909:675 bp | 88.35 | 678 | 15441 | 0 | 795 | 0 | Parnara guttata mitochondrion, complete genome |
| P. glaucus | m | scaffold_9143:575 bp | 85.05 | 562 | 15164 | 1.00E-158 | 562 | 0 | Libythea celtis mitochondrion, complete genome |
| P. glaucus | m | scaffold_20200:447 bp | 87.44 | 398 | 15359 | 7.00E-125 | 449 | 0 | Papilio xuthus mitochondrion, complete genome |
| P. glaucus | m | scaffold_26615:393 bp | 92.79 | 305 | 15290 | 4.00E-122 | 440 | 0 | Hasora anura mitochondrion, complete genome |
| P. glaucus | m | scaffold_7848:599 bp | 88.22 | 365 | 15256 | 1.00E-119 | 433 | 1 | Papilio polytes mitochondrion, complete genome |
| A. niger | m | scaffold_0:31289 bp | 99.12 | 9284 | 31103 | 0 | 16661 | 36 | Aspergillus niger mitochondrion, complete genome |
| A. niger | m | scaffold_572:199 bp | 89.73 | 185 | 92366 | 3.00E-60 | 233 | 0 | Silene noctiflora mitochondrion chromosome 51, complete sequence |
| A. niger | p | scaffold_2:5232 bp | 89.3 | 187 | 156073 | 3.00E-58 | 231 | 0 | Nicotiana otophora chloroplast, complete genome |
| A. niger | m | scaffold_636:174 bp | 84.09 | 176 | 30696 | 1.00E-38 | 161 | 0 | Aspergillus fumigatus mitochondrion, complete genome |
| A. niger | m | scaffold_27:749 bp | 87.97 | 133 | 32827 | 1.00E-34 | 150 | 0 | Aspergillus terreus NIH2624; mitochondrial |
| A. niger | m | scaffold_577:199 bp | 84.47 | 161 | 1829341 | 3.00E-35 | 150 | 0 | Corchorus olitorius mitochondrion, complete genome |
| A. niger | m | scaffold_52:586 bp | 89.72 | 107 | 21449 | 8.00E-31 | 137 | 2 | Arthroderma otae CBS 113480 supercont1.17 mitochondrial scaffold, whole genome shotgun sequence |
| A. niger | m | scaffold_51:587 bp | 95.24 | 84 | 29192 | 4.00E-29 | 132 | 0 | Aspergillus oryzae 3.042 mitochondrion, complete genome |
| A. niger | m | scaffold_41:675 bp | 98.51 | 67 | 33227 | 4.00E-25 | 119 | 0 | Aspergillus nidulans FGSC A4 mitochondrion, complete genome |
| A. niger | m | scaffold_11:823 bp | 94.55 | 55 | 33656 | 4.00E-15 | 86 | 2 | Aspergillus tubingensis mitochondrion, complete genome |
| P. papatasi | m | scaffold_0:15338 bp | 99.54 | 14927 | 15557 | 0 | 27180 | 27 | Phlebotomus papatasi mitochondrion, complete genome |
| P. papatasi | m | scaffold_279:1528 bp | 81.89 | 265 | 92366 | 1.00E-53 | 215 | 1 | Silene noctiflora mitochondrion chromosome 51, complete sequence |
| P. papatasi | m | scaffold_30684:534 bp | 84.85 | 165 | 1829341 | 1.00E-39 | 167 | 0 | Corchorus olitorius mitochondrion, complete genome |
| P. papatasi | p | scaffold_1:4201 bp | 89.91 | 109 | 156073 | 2.00E-29 | 135 | 4 | Nicotiana otophora chloroplast, complete genome |
| P. papatasi | p | scaffold_40807:469 bp | 81.25 | 160 | 183313 | 4.00E-28 | 128 | 0 | Neoizziella asiatica chloroplast, complete genome, isolate J.0154 |
| P. papatasi | p | scaffold_549:1392 bp | 90.77 | 65 | 150510 | 8.00E-15 | 86 | 0 | Dracocephalum palmatum chloroplast, complete genome |
| P. papatasi | m | scaffold_10339:768 bp | 86.89 | 61 | 16004 | 5.00E-09 | 65 | 1 | Pteronarcys princeps mitochondrion, complete genome |
| P. papatasi | m | scaffold_13654:709 bp | 100 | 33 | 77380 | 6.00E-08 | 62 | 0 | Saccharomyces cariocanus strain CBS 7994 mitochondrion, complete genome |
| P. papatasi | m | scaffold_9192:795 bp | 97.06 | 34 | 13909 | 9.00E-07 | 58 | 0 | Strongyloides papillosus isolate LIN mitochondrion, complete genome |
| P. papatasi | m | scaffold_2207:1095 bp | 92.68 | 41 | 52245 | 5.00E-06 | 56 | 0 | Hirsutella minnesotensis strain 3608 mitochondrion, complete genome |

# Additional file 1: Section S2. Extraction of chloroplast from Vittis vinifera (Grape vine)

NOVOPlasty requires a config file, and we updated the config file with the insert sizes and read lengths of this particular dataset. We set the genome range to 100.000 – 300.000 and the K-mer size to 39. The genome range can be changed depending on how much is known about the chloroplast beforehand.

We used a RefSeq sequence as the reference (gi|91983971). This sequence had a length of 160.928 base pairs while our outputted chloroplast had a length of 160.946 base pairs. The total runtime was 12 hours.

Table 2. Blast-results of the assembled mitochondrial sequence against the reference sequence.

| Query | Subject | Identity % | Alignment-length | Mis-matches | Gaps | Query start | Query End | Subject start | Subject end | E-Value | Bit-score |
| --- | --- | --- | --- | --- | --- | --- | --- | --- | --- | --- | --- |
| Contig1 | gi\|91983971\|ref\|NC_007957.1\| | 99.99 | 160945 | 3 | 1 | 1 | 160945 | 2 | 160928 | 0 | 2.97E+05 |
| Contig1 | gi\|91983971\|ref\|NC_007957.1\| | 100 | 26353 | 0 | 0 | 134584 | 160936 | 115493 | 89141 | 0 | 48665 |
| Contig1 | gi\|91983971\|ref\|NC_007957.1\| | 99.93 | 26371 | 1 | 1 | 89140 | 115510 | 160919 | 134567 | 0 | 48579 |

From the blast-results we see that they are very similar. We can also see that there are repeat regions (the second and third line).

Depth comparison:


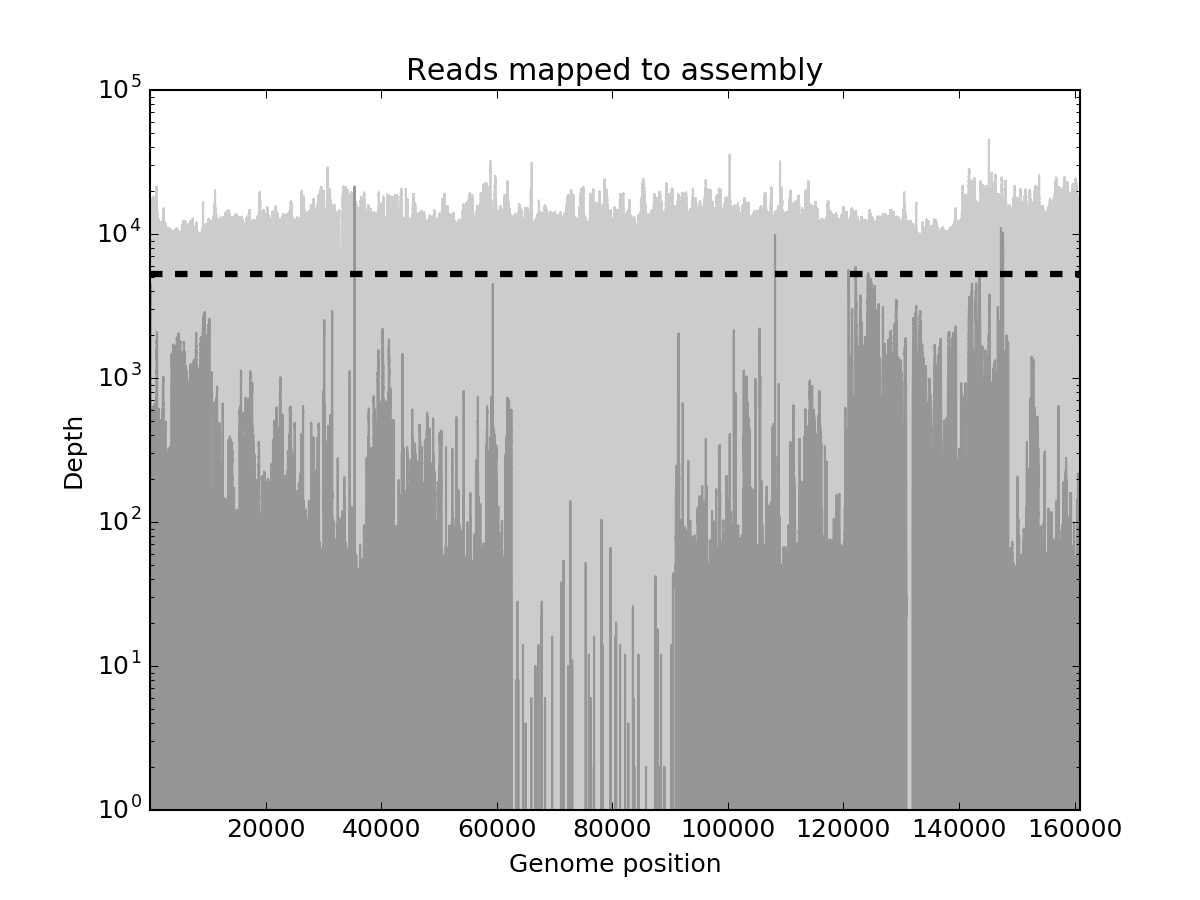


Figure 1. Depth comparison between the mitochondrial sequence (light-grey) and a sub-sequence of the nuclear genome (dark gray) with the same length as the chloroplast sequence. The dashed line is the nuclear depth threshold (ND threshold) that corresponds to the 99.8 percentile of the nuclear depths (dark grey). The depth over the mitochondrial sequence is consistently 10-100 times higher than the depth over the nuclear genome.

# Additional file 1: Section S3. Benchmarking against other methods

| **Sample** | **Ref. length (base pairs)** |  |  |  |  |  |  |  |  |
| --- | --- | --- | --- | --- | --- | --- | --- | --- | --- |
|  |  | Tool | Length (base pairs) | Identity | Gaps | Runtime (hours) | Peak memory usage (GB) | Input reads | Other input |
| *A. melanoleuca* (Giant Panda) | 16,805 | NORGAL | 16,886 | 99.5% | 81 | 10.0 | 48 | **Raw reads** | **None** |
|  |  | MITOBIM | 16,994 | 98.8% | 189 | 0.8 | 1 | Trimmed and interleaved | Reference mitogenome (NC_009492.1) |
|  |  | NOVOPLASTY | 16,827 | 99.1 | 142 | 0 | 53 | Raw reads | Insert size, read length, ref coi (DQ093081.1) |
| *S. japonica* (Japanese Seaweed) | 37,657 | NORGAL | 37,657 | 99.8% | 1 | 8.2 | 47 | **Raw reads** | **None** |
|  |  | MITOBIM | 37,979 | 99.12% | 322 | 2.1 | 1 | Trimmed and interleaved | Reference mitogenome (NC_013476.1) |
|  |  | NOVOPLASTY | 37,657 | 99.8% | 0 | 0 | 38 | Raw reads | Insert size, read length, genome size range, reference COI sequence (JN873222.1) |
| *P. glaucus* (Eastern tiger swallowtail butterfly) | 15,306 | NORGAL | 15,314 | 99.8% | 26 | 11.6 | 38 | **Raw reads** | **None** |
|  |  | MITOBIM | 15,512 | 98.5% | 206 | 1.0 | 3 | Trimmed and interleaved | Reference mitogenome (KR822739.1) |
|  |  | NOVOPLASTY | 15,306 | 100% | 0 | 0 | 34 | Raw reads | Insert size, read length, reference COI sequence (KT286455.1) |
| *A. niger* | 31,103 | NORGAL | 31,353 | 98.7% | 364 | 3.7 | 48 | **Raw reads** | **None** |
|  |  | MITOBIM | 31,747 | 97.8% | 673 | 12.7 | 13 | Trimmed and interleaved | Reference mitogenome (NC_007445.1) |
|  |  | NOVOPLASTY | 31,353 | 98.9% | 315 | 1 | 37 | Raw reads | Insert size, read length, genome size range, reference COI sequence (EF180096.1) |
| *P. papatasi* (Sand fly) | 15,557 | NORGAL | 15,565 | 98.5% | 235 | 13.8 | 46 | **Raw reads** | **None** |
|  |  | MITOBIM | 15,701 | 99.0% | 150 | 0.4 | 1 | Trimmed and interleaved | Reference mitogenome (NC_028042.1) |
|  |  | NOVOPLASTY | 15,567 | 99.9% | 16 | 0 | 33 | Raw reads | Insert size, read length, reference COI sequence (KU659597.1) |

Table 3. Benchmark results. We ran all tests using the recommended settings specified in the available documentation. For all the tests, we do not include the time for data preparation in the final runtime. We used Norgal v1.0, MITOBim v1.8 and NOVOPlasty v2.6.2

# Additional file 1: Section S4. Mitochondrial test data sets

This section shows the results of the different test sets. Not all of them resulted in full mitogenomes. We also ran Norgal against an ancient DNA sample of a horse with limited success most likely due to the very low coverage typical for ancient samples (see figure 10). For the ancient samples, it is necessary to manually set the depth cut-off. Note that the experiment with human reads failed. After mapping the reads to the reference human mitogenome, we saw that the reads only mapped to a region of around 6000 base pairs, suggesting that not all mitochondrial reads are present in the SRA entry.

Table 4. Overview of the benchmarked samples and their results from Norgal. The results marked in **bold** are the ones shown in the figures.

| **SRA accession ID** | **Organism** | **Fig.** | **Result of Norgal** |
| --- | --- | --- | --- |
| SRR1801279 | Aspergillus niger | 2 | **Full circular mitogenome** |
| SRR2089773 | Butterfly (Lerema accius) | 3 | **Full circular mitogenome** |
| SRR2089774 | Butterfly (Lerema accius) | 4 | **Full circular mitogenome** |
| SRR2089775 | Butterfly (Lerema accius) | 5 | **Full circular mitogenome** |
| SRR1707287 | Eastern tiger swallowtail (Papilio glaucus) | 6 | **Full circular mitogenome** |
| SRR543219 | Cucumber (Cucumis sativus) | 7 | Partial chloroplast (8%) and **partial mitochondrial chromosomes 1 (1%)**, chromosome 2 (17%) and chromosome 3 (49%) |
| SRR1997462 | Sand fly (Phlebotomus papatasi) | 8 | **Full circular mitogenome** |
| SRR2015301 | Common grape vine (Vitis vinifera) | 9 | Partial chloroplast (2%) and **partial mitogenome (2%)** |
| ERR1034525 | Ancient Horse (Equus caballus) | 10 | **Partial mitogenome (75%)** |
| SRR1291041 | Human (Homo sapiens) | 11 | Failed |
| SRR958464 | Opossum (Monodelphis domestica) | 12 | **Full circular mitogenome** |
| SRR504904 | Giant panda (Ailuropoda melanoleuca) | 13 | **Full circular mitogenome** |
| SRR942310 | Polar bear (Ursus maritimus) | 14 | **Full circular mitogenome** |
| SRR1993099 | Baker's yeast (Saccharomyces cerevisiae) | 15 | **Partial mitogenome (4%)** |
| ERR1437502 | Baker's yeast (Saccharomyces cerevisiae) | 16 | **Partial mitogenome (59%)** |
| ERR771129 | Baker's yeast (Saccharomyces cerevisiae) | 17 | **Partial mitogenome (13%)** |
| SRR2984940 | Baker's yeast (Saccharomyces cerevisiae) | 18 | **Partial mitogenome (11%)** |
| SRR494422 | Watermelon (Citrullus lanatus) | 19 | Partial chloroplast (38%) and **partial mitogenome (13%)** |
| SRR494432 | Watermelon (Citrullus lanatus) | 20 | Partial chloroplast (20%) and **partial mitogenome (19%)** |
| SRR2043182 | Sea weed (Saccharina japonica C11) | 21 | **Full circular mitogenome** |

The legend for the following graphs are the following:

|  | mtDNA depth |
| --- | --- |
|  | Nuclear depth |
|  | Cut-off |

The light grey area shows the depth over the longest potential mitochondrial sequence and the dark area shows depth over a subsection of the nuclear genome that is as long as the potential mitochondrial sequence or shorter. The dashed line shows the cut-off that was either set by NORGAL or manually by the user.


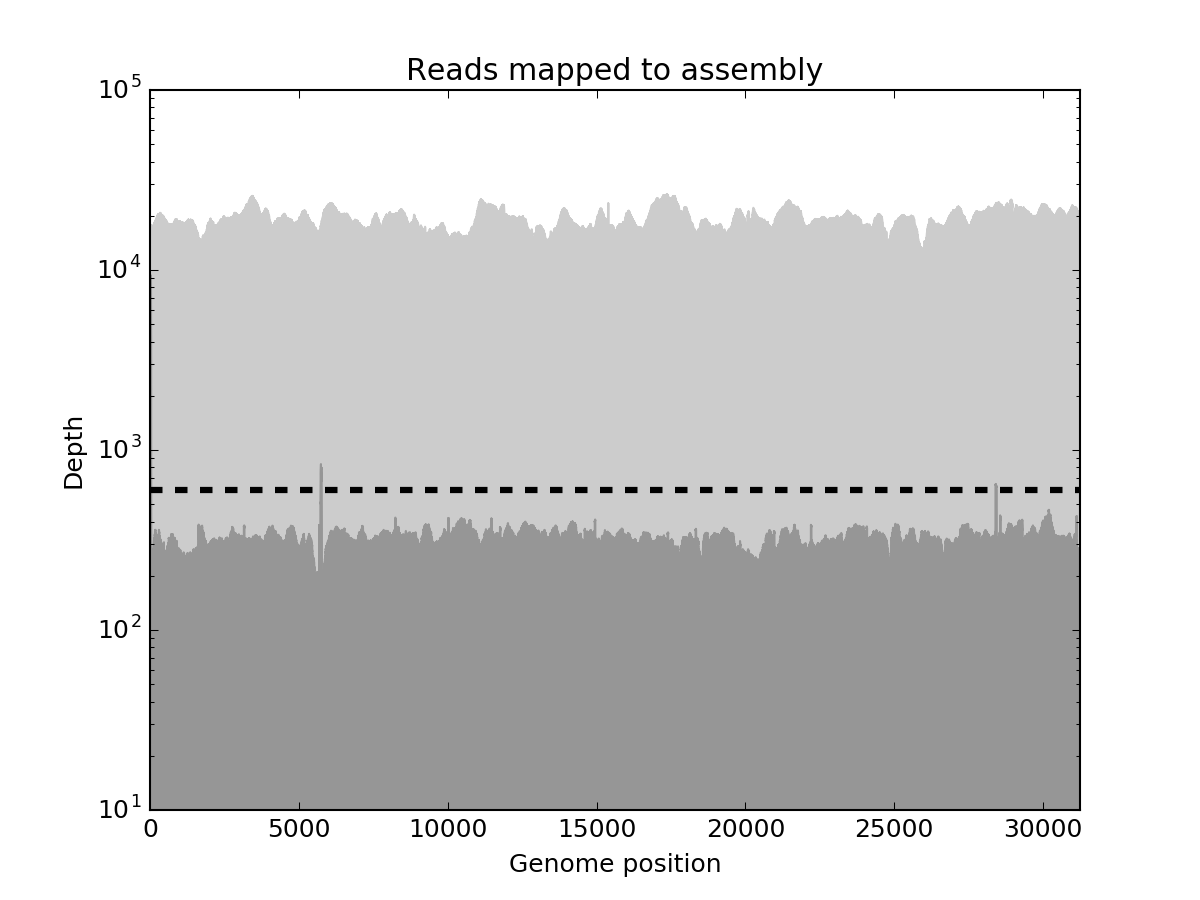


Figure 2. Aspergillus niger, SRR1801279


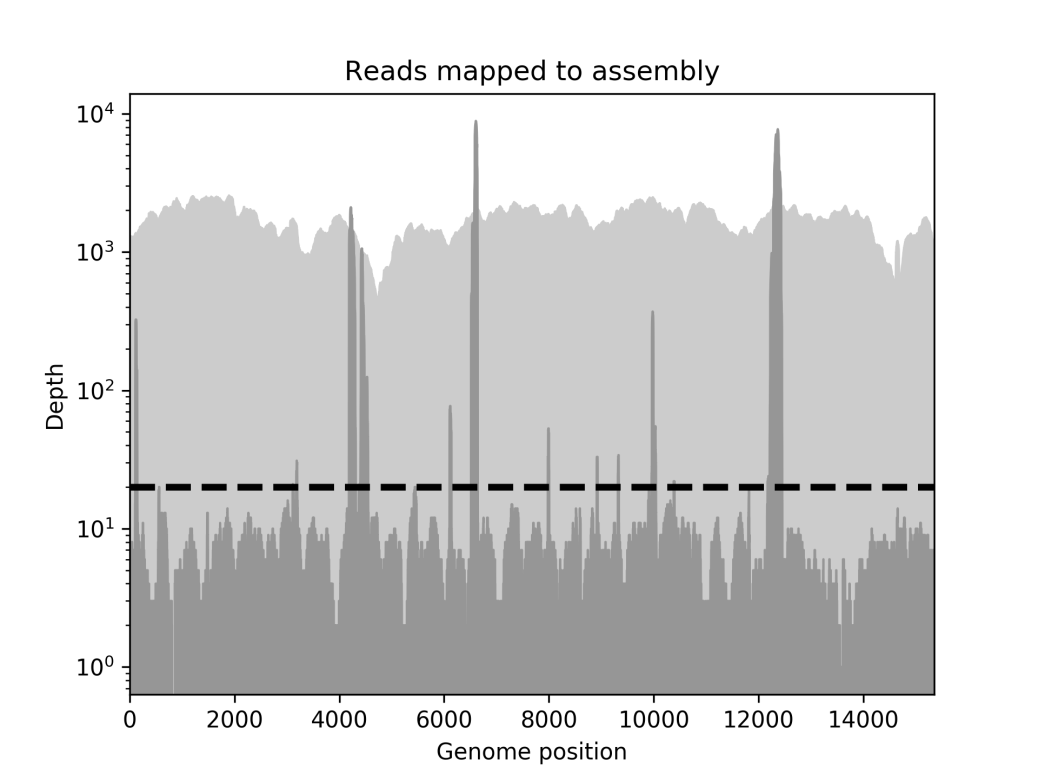


Figure 3. Butterfly (Lerema accius), SRR2089773


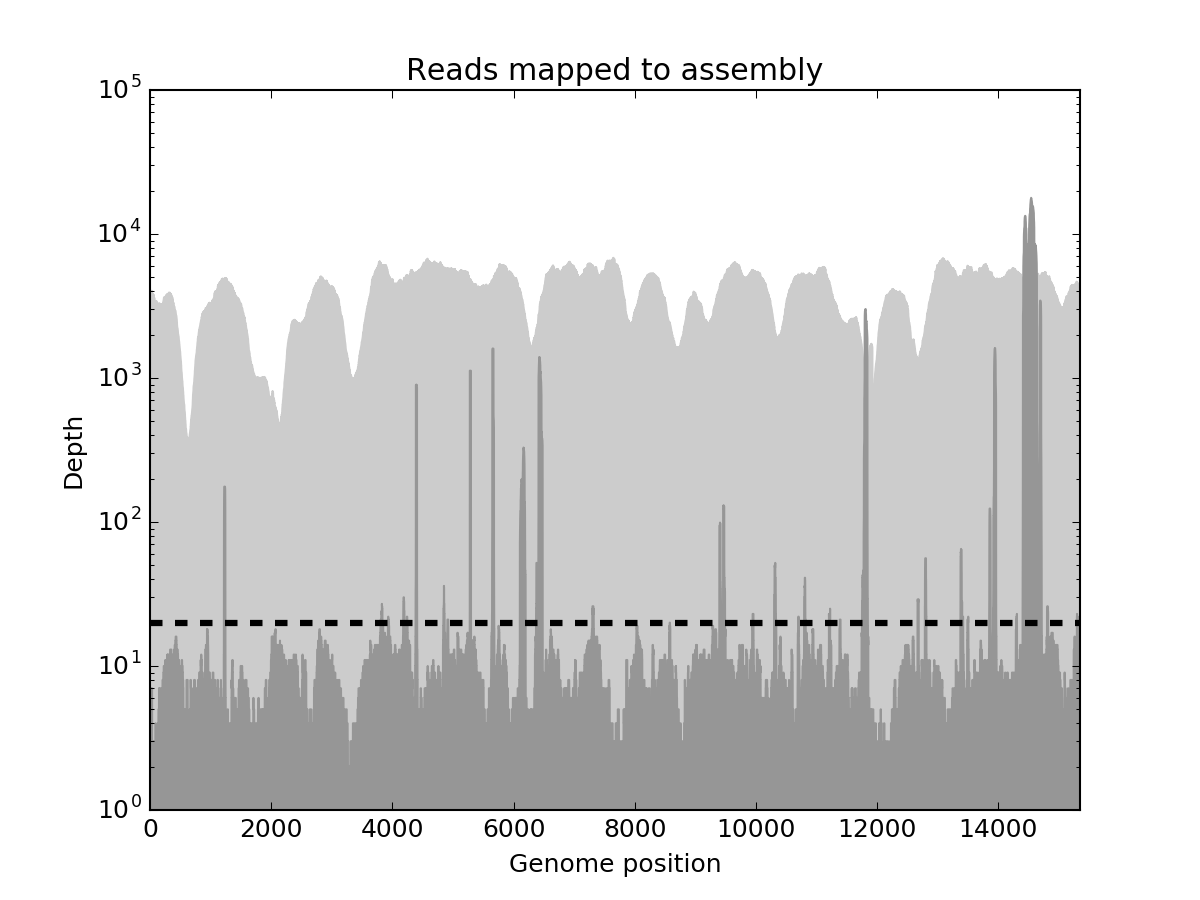

Figure 4. Butterfly (Lerema accius), SRR2089774


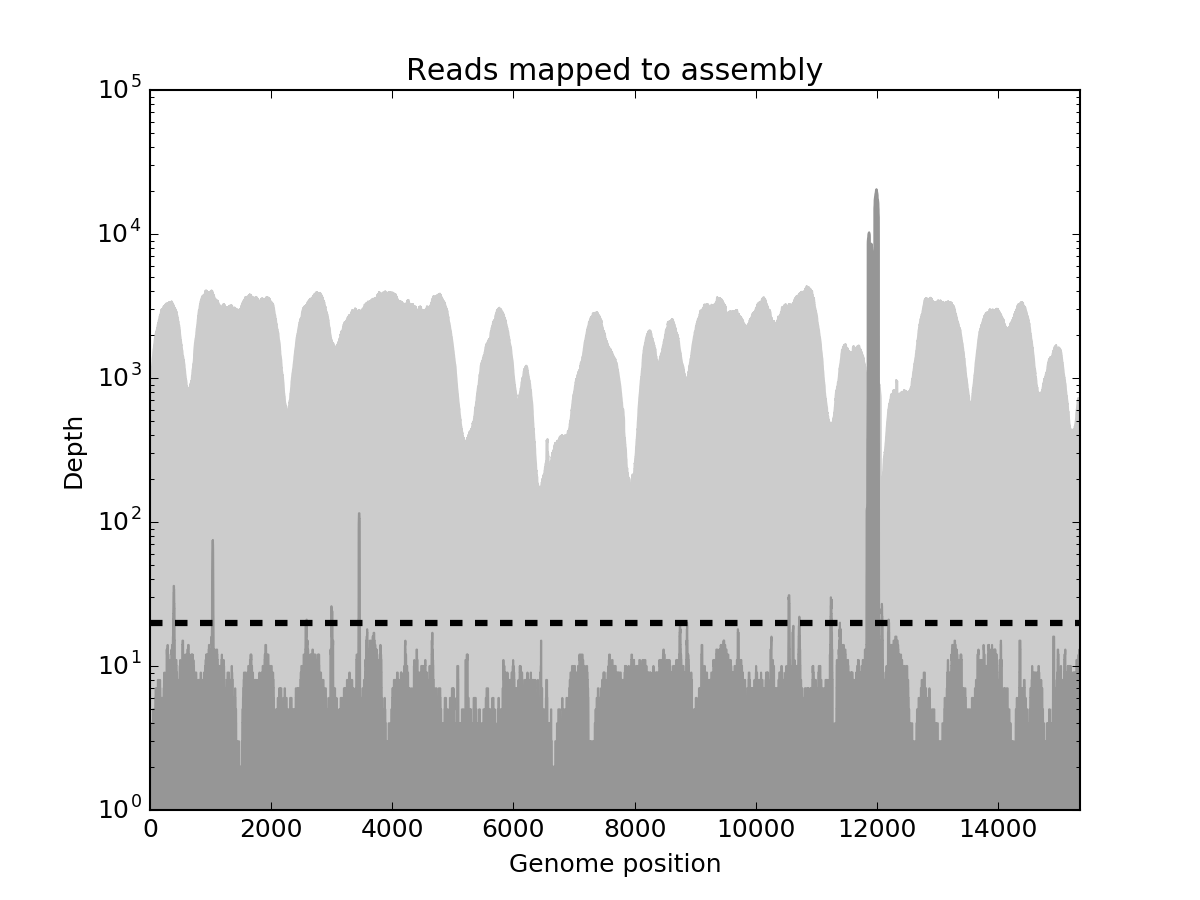


Figure 5. Butterfly (Lerema accius), SRR2089775


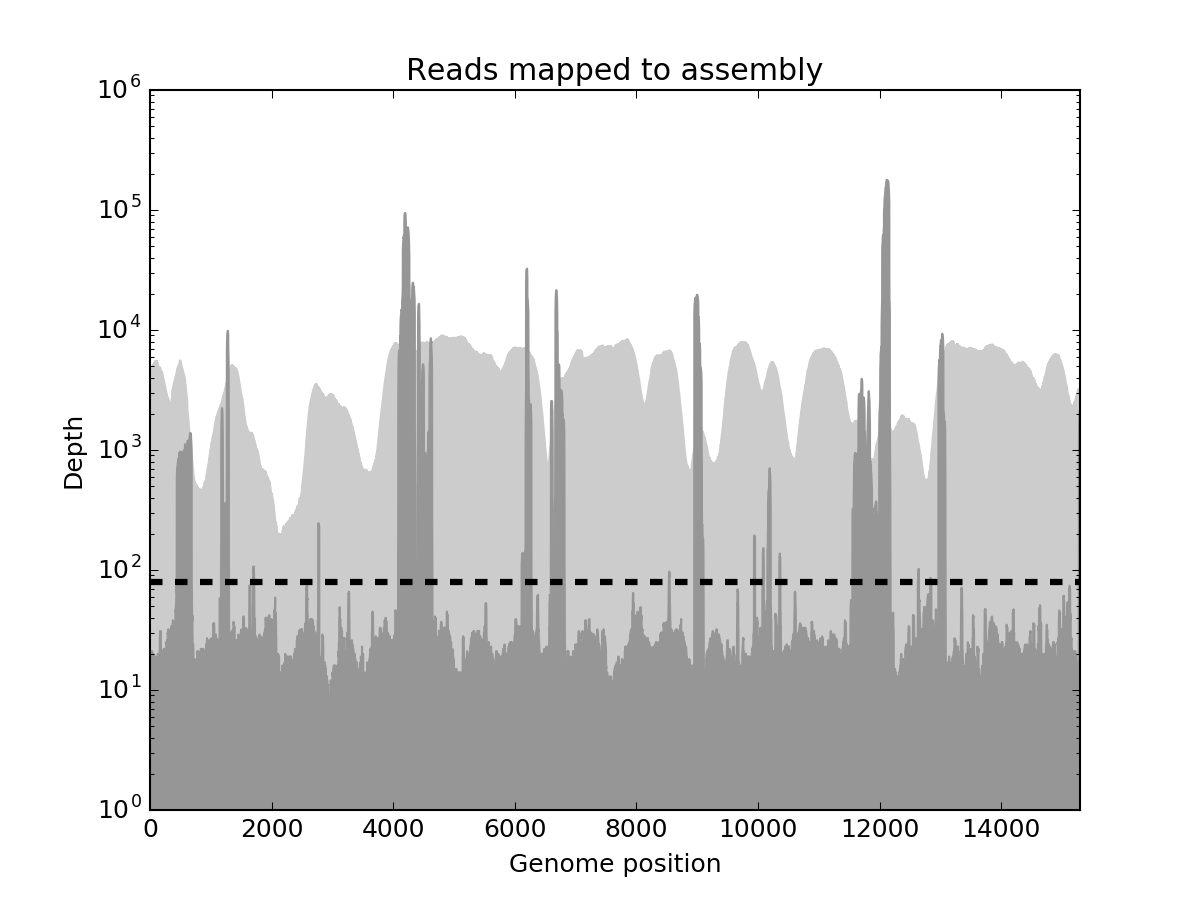

Figure 6. Butterly (Papilio glaucus), SRR1707287


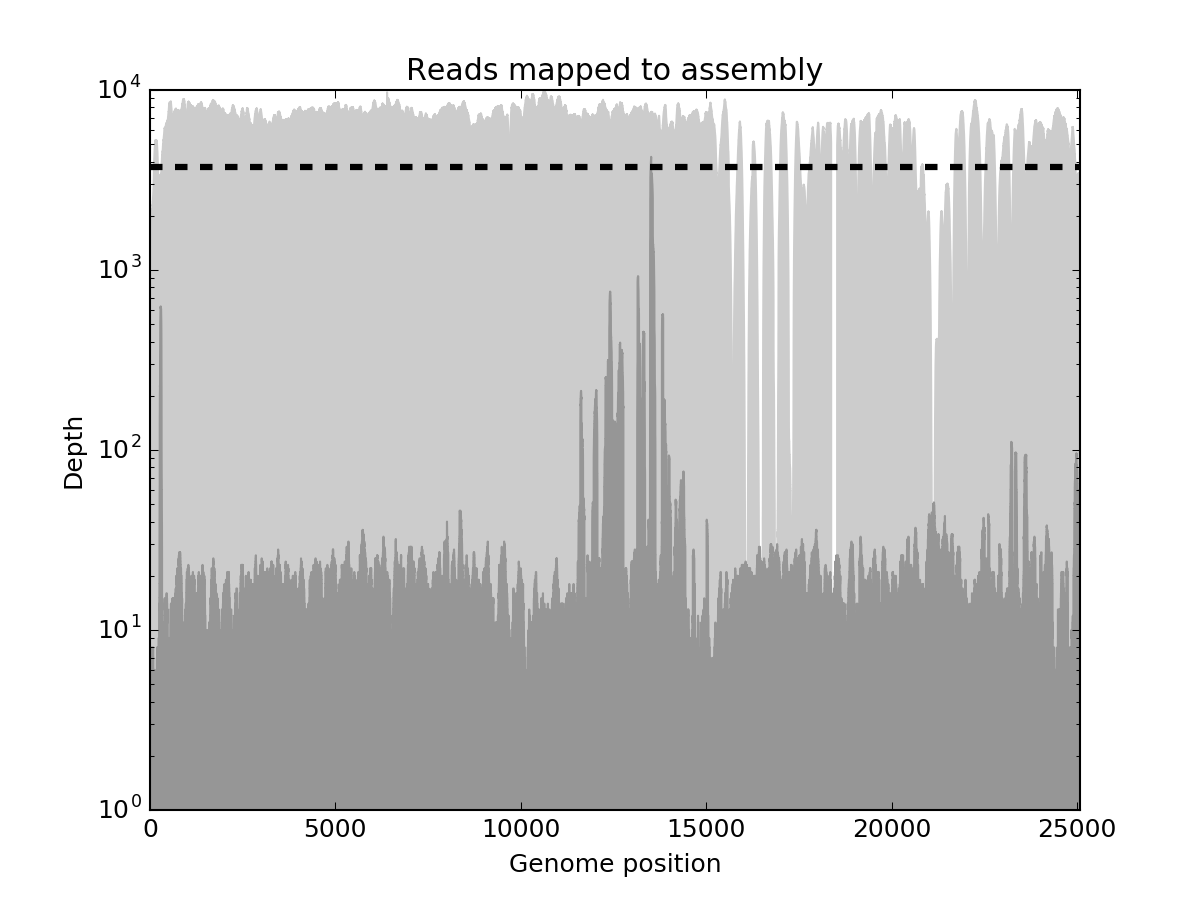


Figure 7. Cucumber (Cucumis sativus), SRR543219


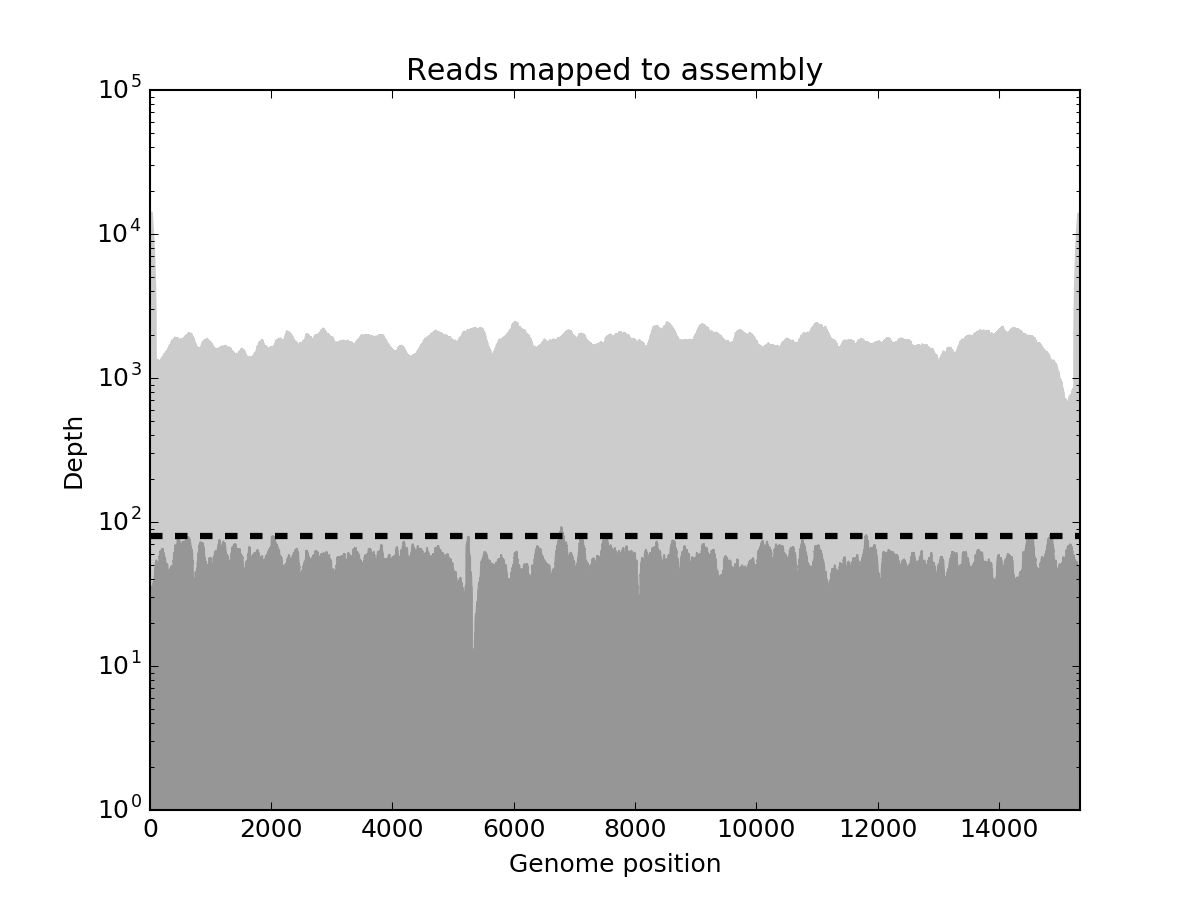


Figure 8. Sand fly (Phlebotomus papatasi), SRR1997462


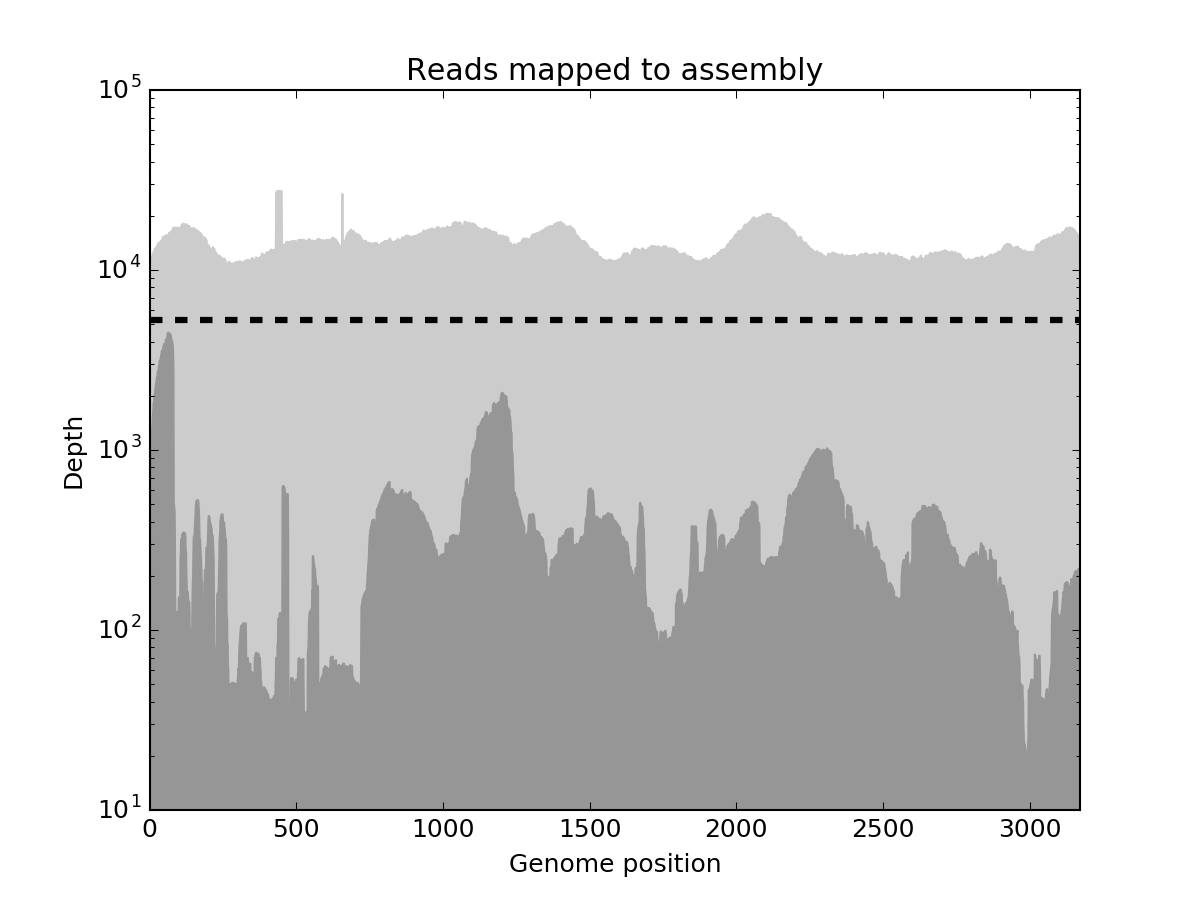


Figure 9. Common grape vine (Vitis vinifera), SRR2015301


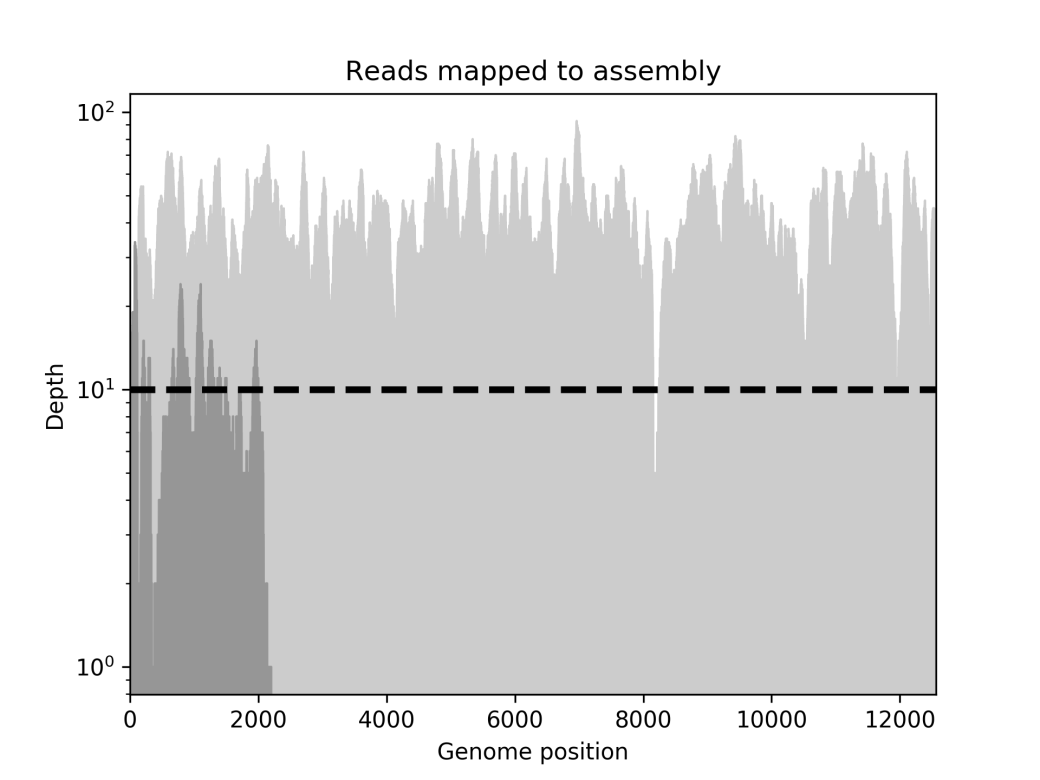


Figure 10. Ancient Horse (Equus caballus), SRR899957


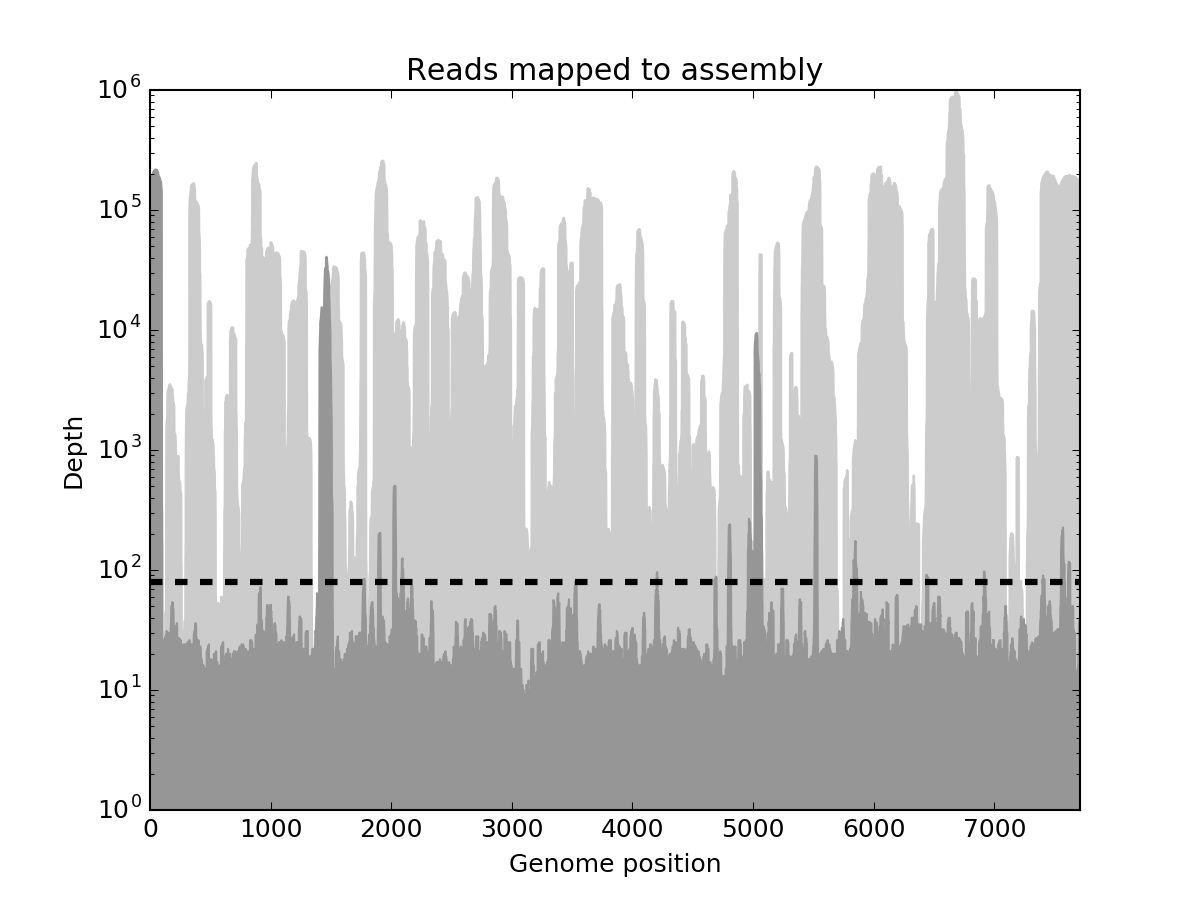


Figure 11. Human (Homo sapiens), SRR1291041


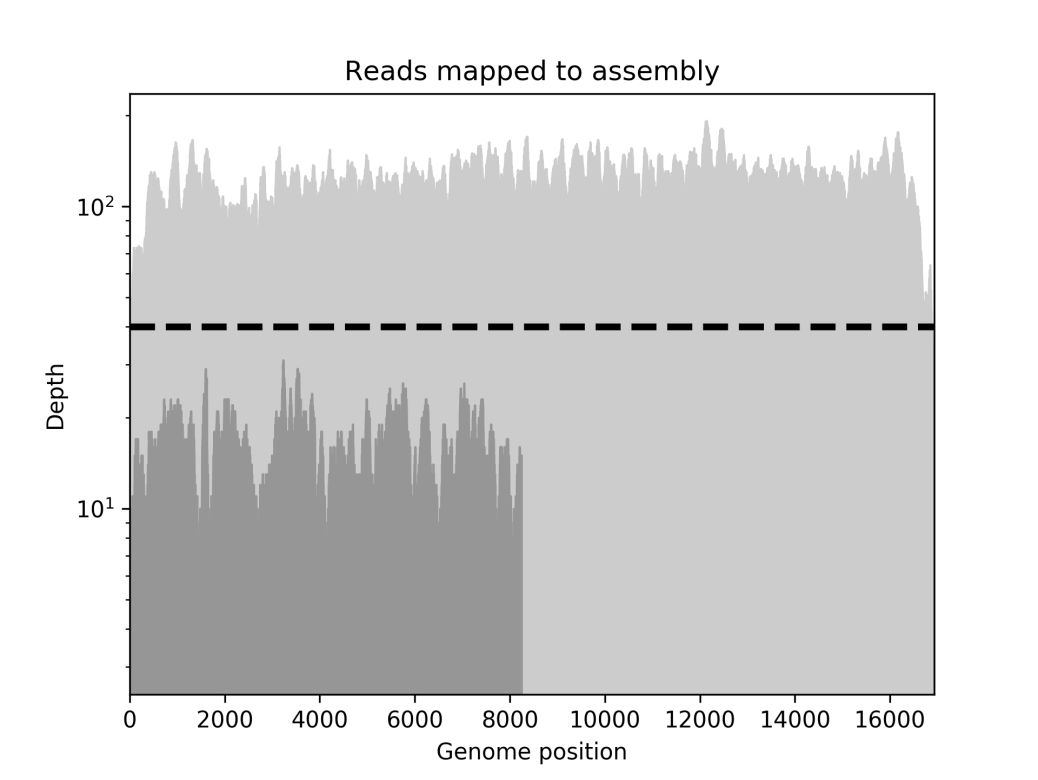


Figure 12. Opossum (Monodelphis domestica), SRR958464


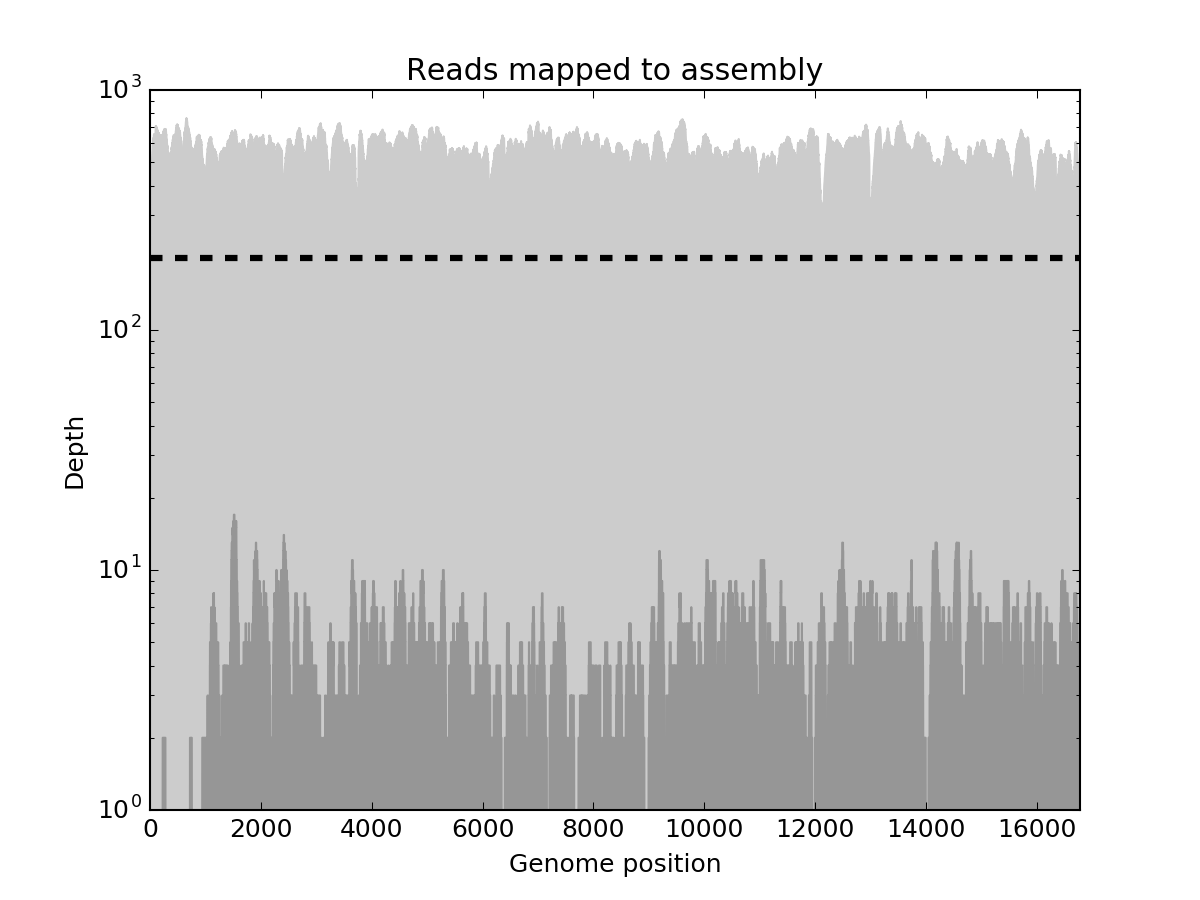


Figure 13. Giant panda (Ailuropoda melanoleuca), SRR504904


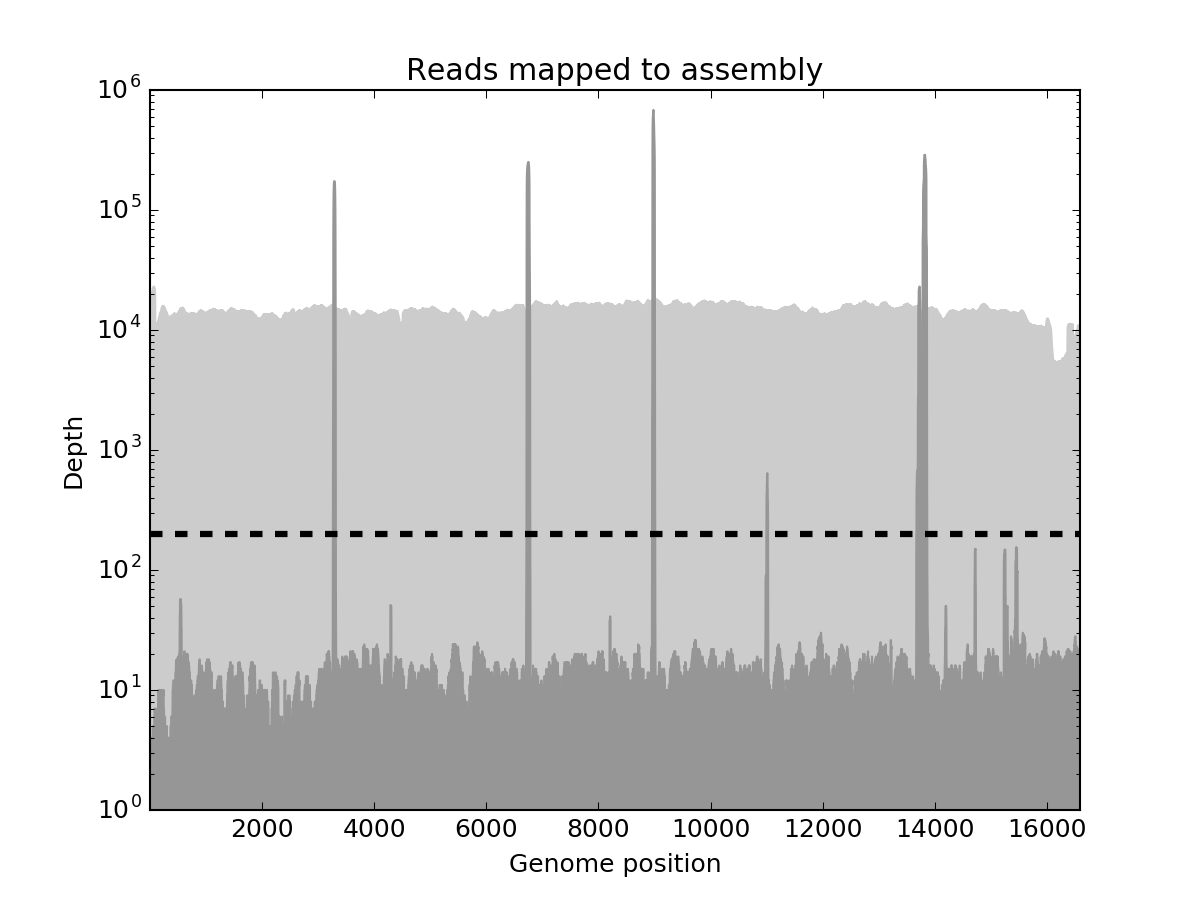


Figure 14. Polar bear (Ursus maritimus), SRR942310


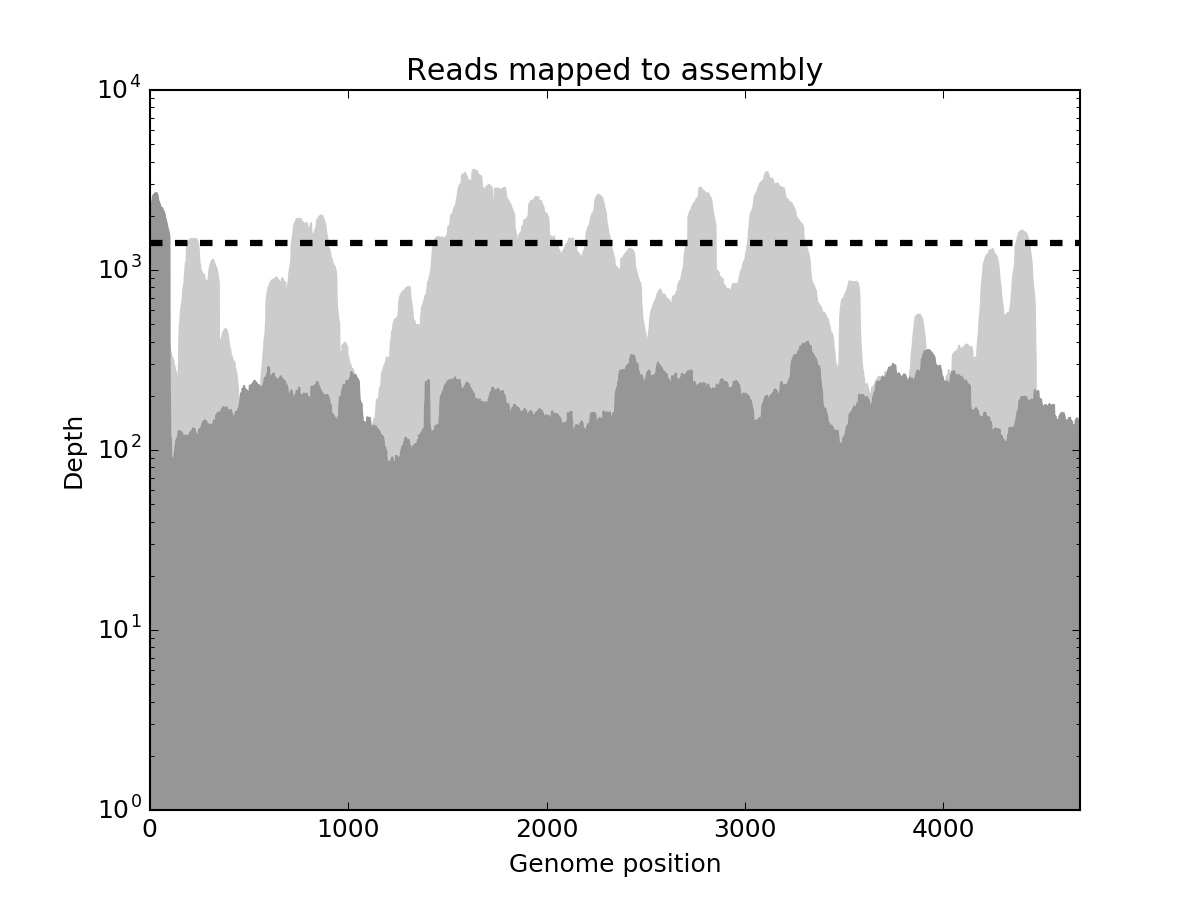


Figure 15. Baker's yeast (Saccharomyces cerevisiae), SRR1993099


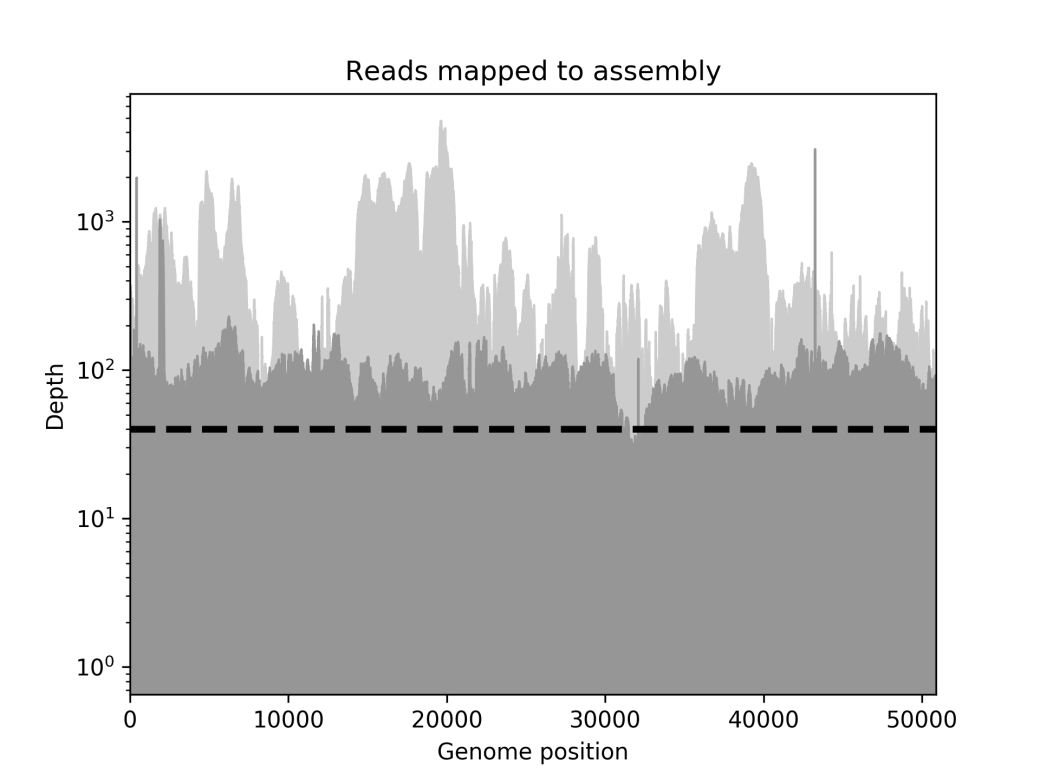


Figure 16. Baker's yeast (Saccharomyces cerevisiae), ERR1437502


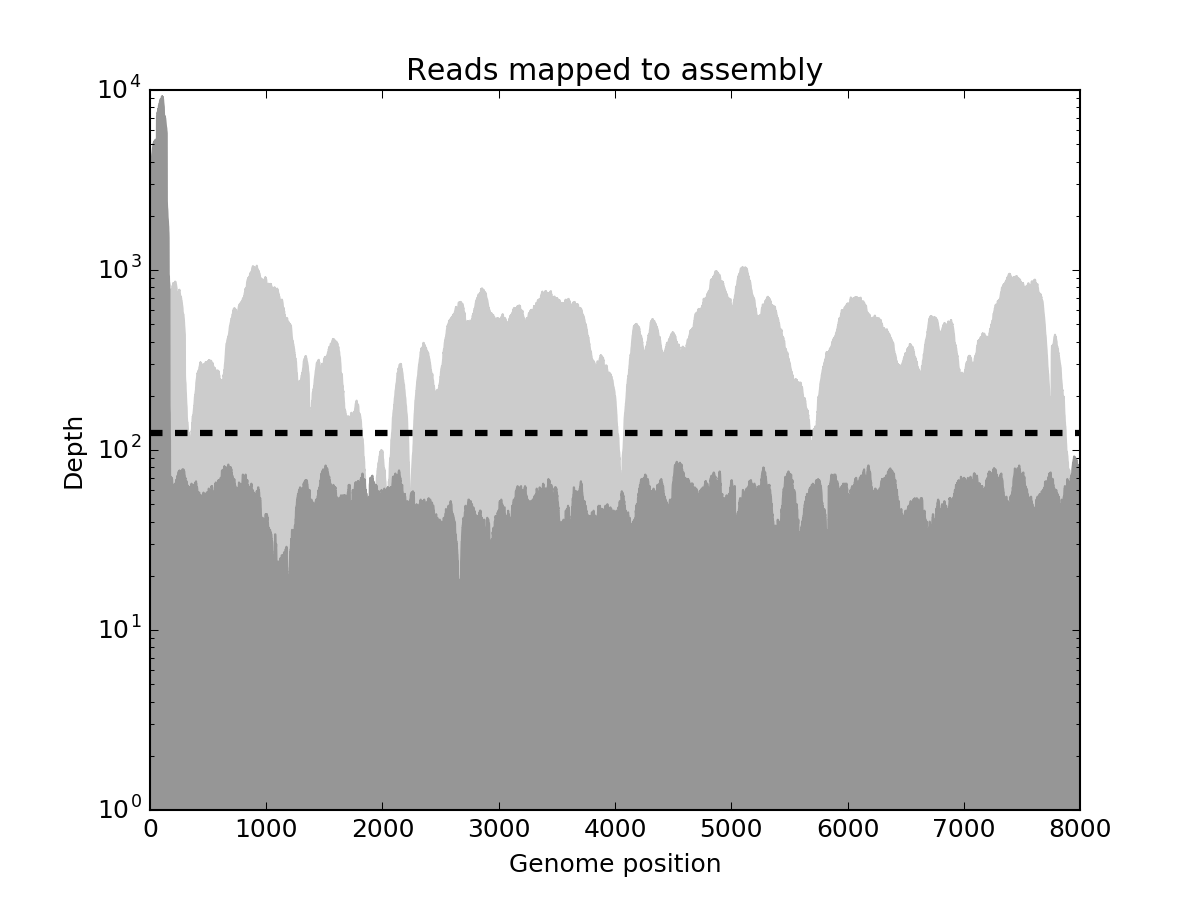


Figure 17. Baker's yeast (Saccharomyces cerevisiae), ERR771129


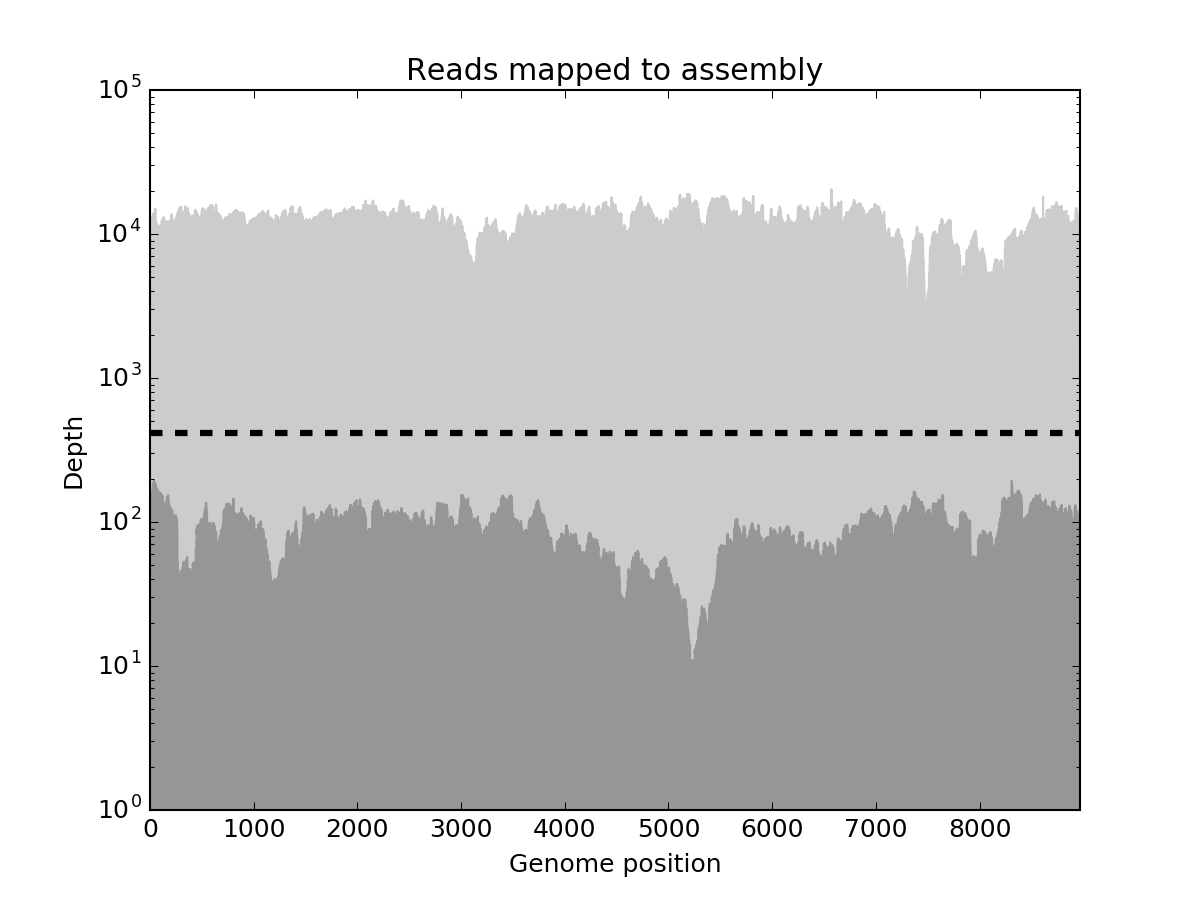


Figure 18. Baker's yeast (Saccharomyces cerevisiae), SRR2984940


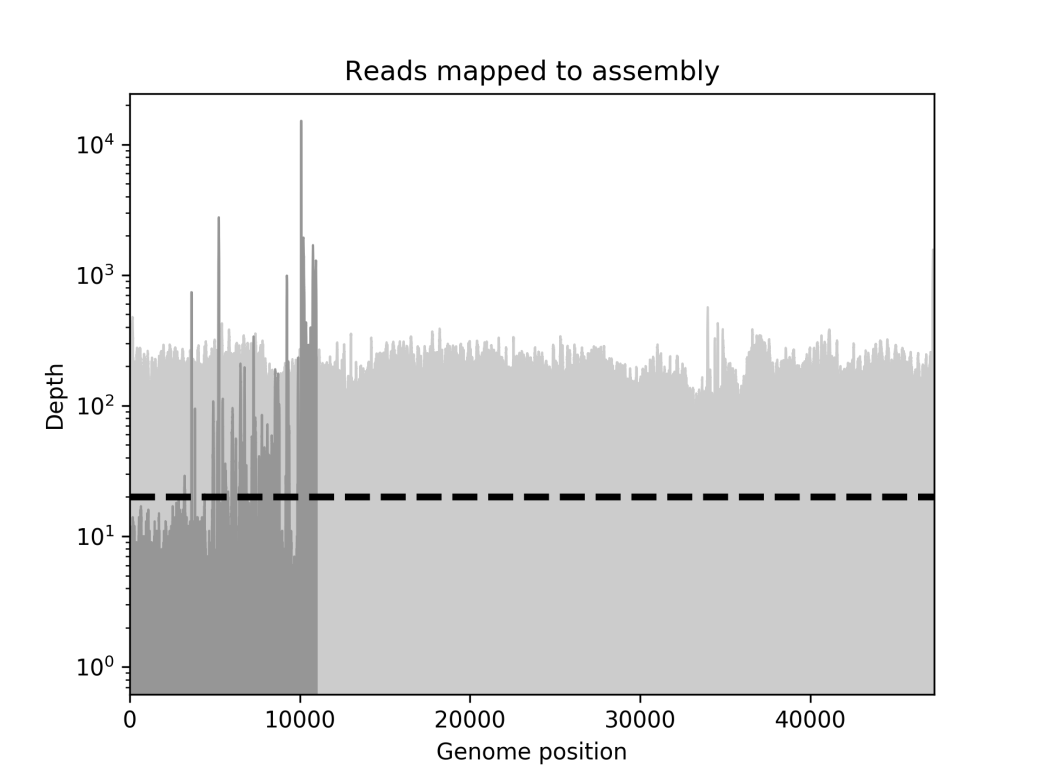


Figure 19. Watermelon (Citrullus lanatus), SRR494422


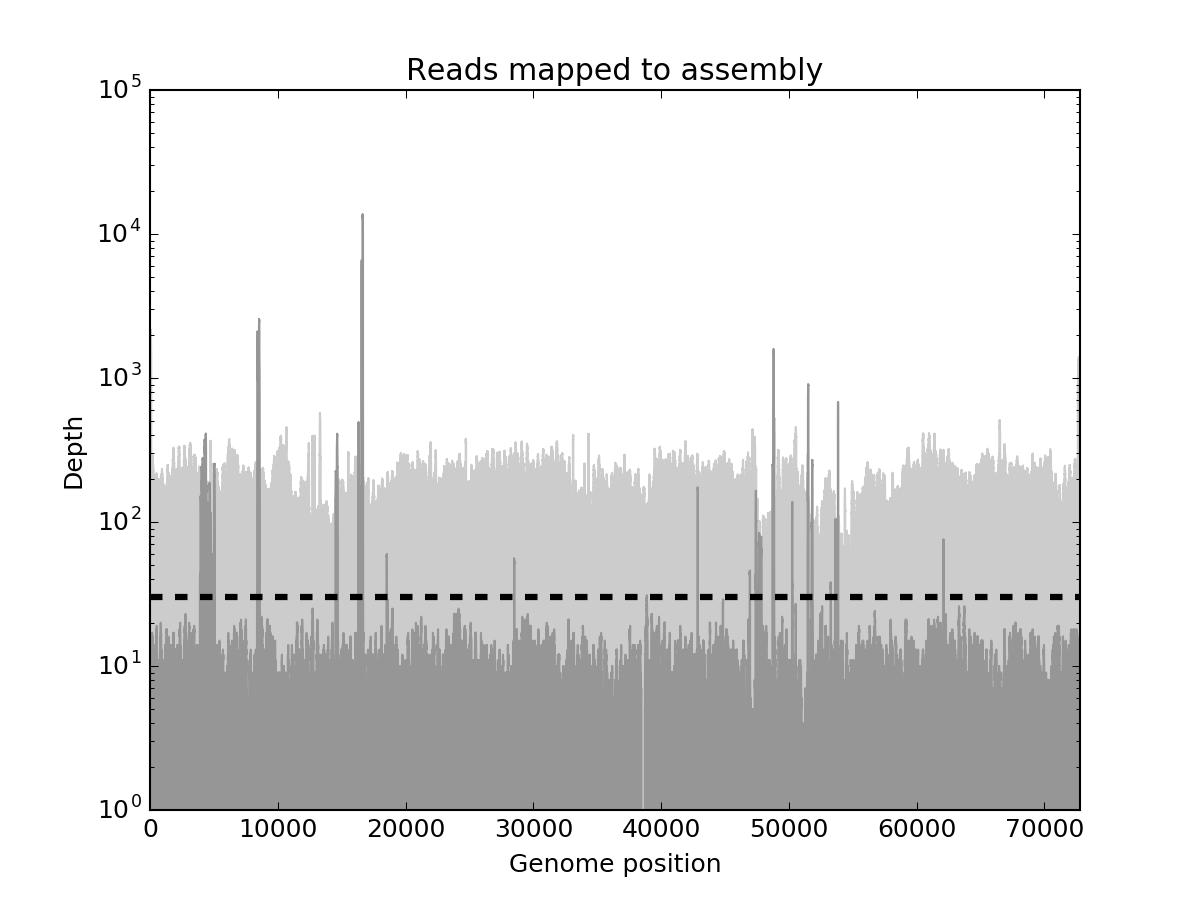


Figure 20. Watermelon (Citrullus lanatus), SRR494432


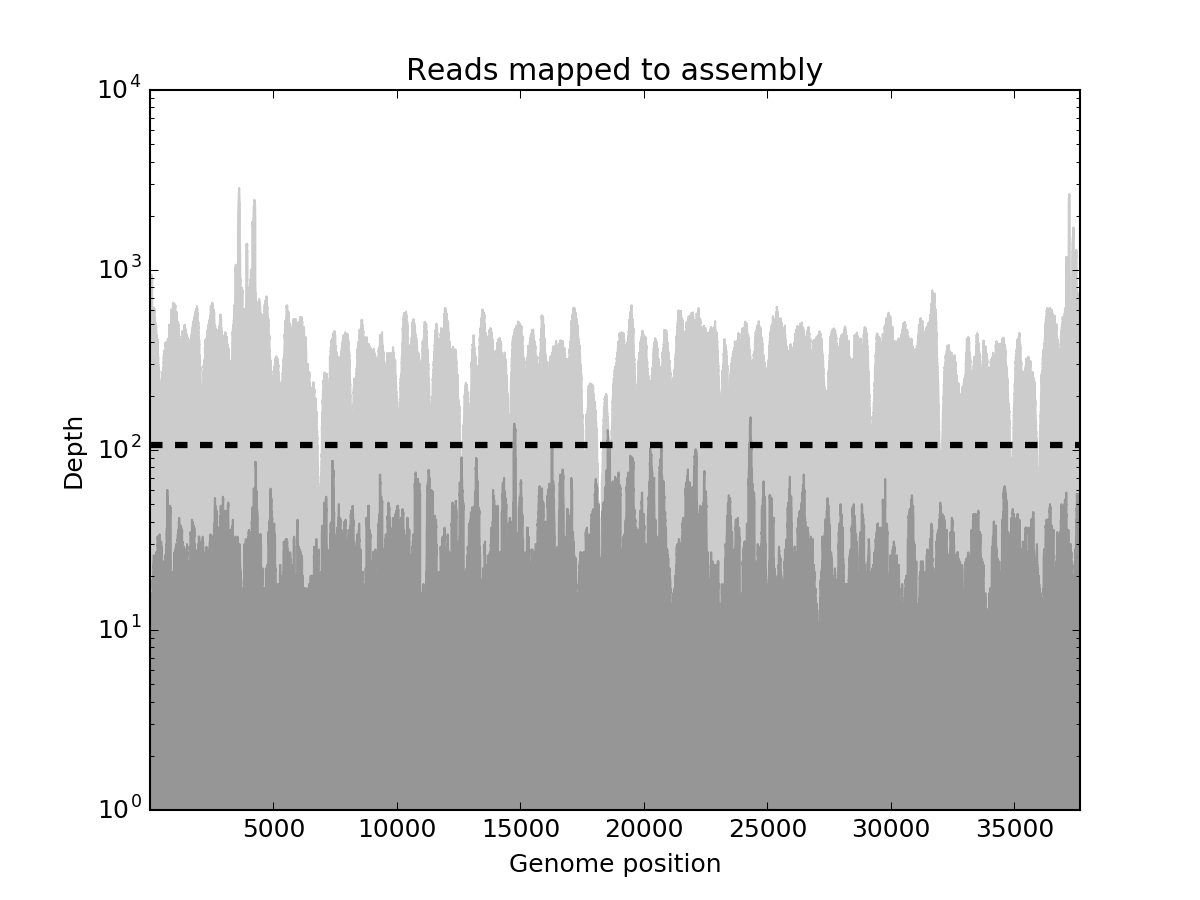


Figure 21. Sea weed (Saccharina japonica C11), SRR2043182
